# Quantifying redundancies and synergies with measures of inequality

**DOI:** 10.1371/journal.pone.0313281

**Published:** 2024-11-20

**Authors:** Tobias Mages, Christian Rohner

**Affiliations:** Department of Information Technology, Uppsala University, Uppsala, Sweden; University of Salerno: Universita degli Studi di Salerno, ITALY

## Abstract

Inequality measures provide a valuable tool for the analysis, comparison, and optimization based on system models. This work studies the relation between attributes or features of an individual to understand how redundant, unique, and synergetic interactions between attributes construct inequality. For this purpose, we define a family of inequality measures (f-inequality) from f-divergences. Special cases of this family are, among others, the Pietra index and the Generalized Entropy index. We present a decomposition for any f-inequality with intuitive set-theoretic behavior that enables studying the dynamics between attributes. Moreover, we use the Atkinson index as an example to demonstrate how the decomposition can be transformed to measures beyond f-inequality. The presented decomposition provides practical insights for system analyses and complements subgroup decompositions. Additionally, the results present an interesting interpretation of Shapley values and demonstrate the close relation between decomposing measures of inequality and information.

## Introduction

Understanding and analyzing inequalities within a population is a central research focus in economics and the social sciences. Measures of inequality provide quantitative insights into the existence and extent of inequality. These measures evaluate the distribution of an *indicator variable*, a non-negative value representing the property of interest for each individual. For example, we may use the disposable income of each individual in a population as indicator variables, and the Gini coefficient as inequality measure to evaluate its distribution.

To reveal the underlying structure and contributing factors of inequality, it is necessary to decompose inequality by forming subgroups within the population. Such decompositions deepen our comprehension of social inequalities and extend to fields like engineering, where they can aid in analyzing and optimizing systems. For this reason, our work focuses on two key questions to provide deeper insights for such analyses: how can inequality be quantified and decomposed?

First, we introduce a family of inequality measures (*f*-inequality), which generalizes existing measures like the Pietra index, Generalized Entropy index, or Atkinson index (see Section “Defining f-inequality”). These measures are derived from f-divergences and deepen the relationship between information theory and inequality measures—a connection previously established by Theil [1] and Shorrocks [2].

**Fig 1:**
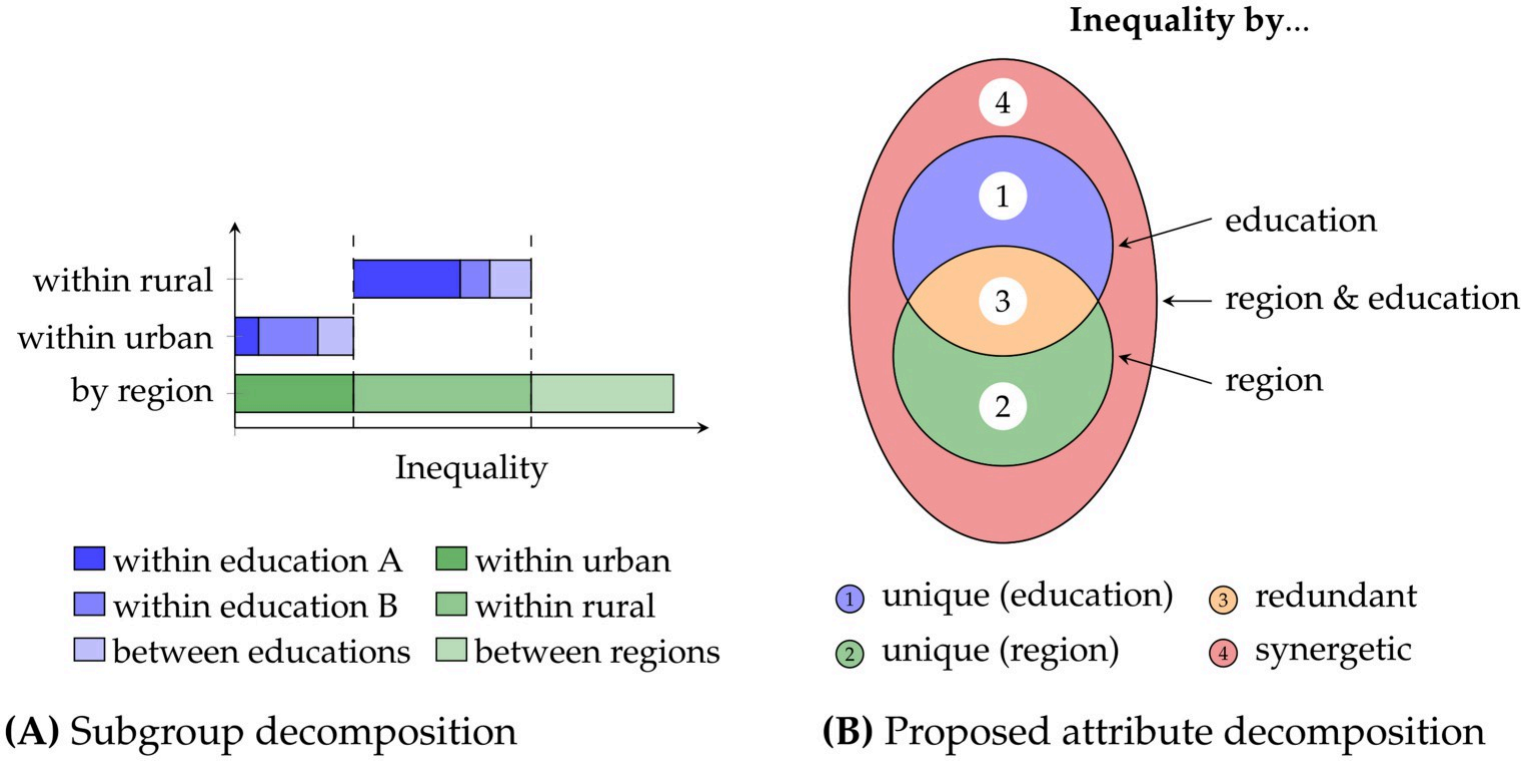
Intuition for the relation between a subgroup decomposition and the proposed attribute decomposition. Consider a population where individuals have the attributes of education (type A/type B) and living region (rural/urban): (A) A subgroup decomposition can provide detailed insights for the possible values of an attribute, such as the region being rural or urban. However, it does not provide insights into the dynamics between attributes. (B) The proposed attribute decomposition provides detailed insights into the interaction between attributes, such as redundant and synergetic effects between educations and regions. However, it does not provide insights for particular attribute values, such as rural and urban regions. Therefore, subgroup and attribute decompositions complement each other.

Second, we present a novel decomposition method to study interactions between the attributes of individuals. Using the example in Fig 1, every individual may have attributes, such as an education type and region, besides its indicator value. We want to understand how redundant, unique, or synergetic interactions between these attributes characterize the inequality in the distribution of the indicator variable. Inspired by recent advancements in information theory [3, 4], the presented decomposition is constructed by relating the lattice formed by the Atkinson criterion to the desired decomposition model. This provides a practical operational interpretation and an intuitive set-theoretic behavior as illustrated in Fig 1B. We demonstrate how this decomposition is achieved for any *f*-inequality (see Section “Decomposing f-inequality”) and extend the approach to their transformations, such as the Atkinson index (see Section “Decomposing the Atkinson index”).

### Related work

The quantification and decomposition of inequality have mainly been driven by economic research [1, 5–9]. An established framework within this area is the *subgroup decomposition* [10–13]: As indicated by Eq (1) and Fig 1A, this framework considers a particular partitioning of the population into subgroups. The framework aims to decompose the total inequality into the inequality between subgroups and the inequality within subgroups.

$$\begin{array}{c} \text{Total}\;\text{Inequality}=\text{(Inequality}\;\text{between}\;\text{subgroups)}+\sum_{\text{subgroup}\;\in \;\text{Partition}}\text{(Inequality}\;\text{within}\;\text{subgroup)} \end{array}$$ (1)

As visualized in Fig 1A, this can provide detailed insights into the attribute values that characterize a subgroup. For example, we can see inequality with respect to the different regions and industries but do not clearly see the interactions between industries and regions.

This work presents a complementing *partition decomposition* or *attribute decomposition*, as visualized in Fig 1B. We decompose inequality into different population partitionings to characterize the dynamics between attributes. The resulting decomposition provides insights into how inequality is constructed from redundant, unique, and synergetic effects between attributes, as indicated by Eq (2).

$$\begin{array}{c} \begin{array}{cc} \text{Total}\;\text{Inequality}= & \;\text{(redundant}\;\text{between}\;\text{attributes)}+\text{(unique}\;\text{to}\;\text{first}\;\text{attribute)}\\ & \;\;+\text{(unique}\;\text{to}\;\text{second}\;\text{attribute)}+\text{(synergetic}\;\text{between}\;\text{attributes)} \end{array} \end{array}$$ (2)

In summary, a subgroup decomposition studies the interactions between subgroups for a particular population partitioning. An attribute decomposition studies the interactions between possible partitionings of the population based on the attributes of individuals.

## Background, preliminaries and examples

**Remark 1**. *Throughout this work, we assume access to some empirical/estimated/known distribution of the indicator variable. All concepts within this work can be described by probability distributions or a finite set of given samples. Since common inequality measures are typically expressed in terms of the latter, we provide all definitions in the same format. This also enables the discussion of small and intuitive examples. However, all presented definitions can be adjusted for the computation on a given probability distribution rather than a given set of samples. Methods for estimating the relevant distributions are discussed among others in* [14–17].

### Definitions and notation

The following Example 1 provides an overview and intuition for the used notation introduced below (Notation 1/Definition 1). The example contains all required notation and concepts for the remaining work.

**Notation 1**.

- *We use subscripts to distinguish variable names, such as* $s_{1},s_{2}\in R_{\geq 0}$ .
- *We notate the power set as* P(·) *and the set of all multisets as* $P_{M}\left(\cdot \right)$ .
- *We notate the Cartesian product of two sets by* **A** × **B**.
- *We notate the n-ary Cartesian product for a set of sets by* C(·) .
- *We notate the additive union of multisets as* **A** ⊎ **B**.
- *We reserve the variable n* ≥ 1 *for the total number of features/attributes of each individual*.
- *We indicate the set of values for a categorical feature/attribute as* $A_{i}$ *with i* ∈ {1, .., *n*}.
- *We write the function τ*(*i*, ⋅) *to access the i*-*th elements of a tuple starting from zero*.
  *For example τ*(*i*, (0, .., *i*)) = *i*. *This notation is only found in Definition 1 and Assumption 1*.
- *We indicate the multiset containing y*-*times the value x by* {*x*}^*y*^ *such as* {1}^3^ = {1, 1, 1}. *This notation is only found in* Eq (4a).

**Definition 1**.

- *An **individual** is a tuple* $\rho \in (R_{\geq 0}\times A_{1}\times \ldots \times A_{n})$ . *The first element* $\tau \left(0,\rho \right)\in R_{\geq 0}$ *represents its non-negative indicator variable. The remaining elements* (0 < *i* ≤ *n*) *represent its categorical features/attributes* $\tau \left(i,\rho \right)\in A_{i}$.
- *We define a **model** as multiset of individuals* $M\in P_{M}\left(R_{\geq 0}\times A_{1}\times ...\times A_{n}\right)$ . *The distribution of indicator values and attributes may be obtained from empirical data and/or estimations. We reserve the symbol* **M** *throughout this work to indicate a model*.
- *We define a **population*** $S\in P_{M}\left(R_{\geq 0}\right)$ *as multiset of indicator values. Throughout this work, we reserve the symbol* **S** *for multisets of indicator values and note the average indicator value (arithmetic mean) of* **S** *by* S¯≔1S·∑s∈Ss .
- *We define a **subgroup** by a function ϑ*(**B**, **M**) *that takes a set of attribute indices and values* (*i*, *a*) ∈ **B** *with a model* **M** *and returns a population by selecting the indicator values of individuals that satisfy all given attributes*.
  $$\begin{array}{c} \vartheta (B,M)≔\{\tau (0,\rho )\;:\;\rho \in M\;\;\text{and}\;\;(\forall (i,a)\in B)[\tau (i,\rho )=a]\} \end{array}$$ (3)
- *We define a **partitioning** of a model by a function* Γ(**a**, **M**) *that takes a set of attribute indices i* ∈ **a** *and a model* **M** *and returns a population. Each distinct subgroup from the considered attributes shall be represented by its size and cumulative indicator value. As it can be seen from Section “Lorenz curves and their ordering”, this is (Lorenz) equivalent to representing each individual ρ* ∈ **M** *by the average indicator value of its subgroup. Therefore, we define a function* *ϖ*(**B**) *that takes a subgroup generator* **B** *and returns a population where each individual is represented by the average indicator value of the corresponding subgroup ϑ*(**B**, **M**).
  $$\begin{array}{cccc} \varpi (B)≔ & {\left\{\bar{S}\right\}}^{\left|S\right|} & & \;where:\;S=\vartheta (B,M) \end{array}$$ (4a)
  $$\begin{array}{cccc} \Gamma (a,M)≔ & \underset{B\in C}{⊎}\;\varpi \left(B\right) & & \;where:\;C=C(\{\left\{i\right\}\times A_{i}\;:\;i\in a\}) \end{array}$$ (4b)
- *We notate an inequality measure as function* $I:P_{M}\left(R_{\geq 0}\right)\to R_{\geq 0}$ *that assigns a non-negative real value to any population*.

**Assumption 1**. *Throughout this work, we assume that indicator values are non-negative (*∀*ρ* ∈ **M**: *τ*(0, *ρ*) ≥ 0*) and that at least one individual has a non-zero indicator value (*∃*ρ* ∈ **M**: *τ*(0, *ρ*) > 0).

**Example 1**. *To give an intuition for the use of Definition 1, consider the minimal example of analyzing the income of a population where each individual has an education type (A/B) and region (urban/suburban/rural)*.

- *The two attributes (n = 2) of individuals are their education type* $A_{1}=\left\{A,B\right\}$ *and region* $A_{2}=\left\{urban,suburban,rural\right\}$.
- *Let the indicator variable of individuals be their equivalised disposable income per year in Swedish krona thousands*.
- *Let the system model consist of four individuals:*
  **M** = {(300, *A, urban*), (500, *B, suburban*), (300, *B, suburban*), (100, *A, rural*)}.
  *Each entry represents an individual by: (disposable income, education type, region). The first entry always represents the indicator value and the remaining attributes are used to partition the population*.
- *The population ϑ*({(1, *B*)}, **M**) = {500, 300} *is the subgroup of individuals with the first attribute (education type) being B. This subgroup has an average indicator value of* 400.
- *The partition on the first attribute (education type) gives the population* Γ({1}, **M**) = {200, 400, 400, 200}. *The partition on no attribute gives a uniform distribution* Γ(*ϕ*, **M**) = {300, 300, 300, 300}, *since* **C** = {ϕ} *in* Eq (4b) *and ϑ*(*∅*, **M**) *returns the indicator value of all individuals*.
- *Note that we refer with ‘total inequality’ to the inequality between possible subgroups and thus between distinguishable individuals based on all given attributes I*(Γ({1, .., *n*}, **M**)). *The partition on all attributes in this example is* Γ({1, 2}, **M**) = {300, 400, 400, 100} *since the second and third individuals are not distinguishable by their attributes (education type: B/region: suburban). This issue can be resolved by adding a unique indentifier (ID) as third attribute* $A_{3}$.

### Measuring inequality

Measuring inequality creates an order in which distributions or populations are considered more unequal than others. This is commonly achieved by defining measures that preserve the following property [5, 6, 18]: a transfer which reduces the difference between two individuals’ indicator value (Pigou-Dalton transfer) can only decrease inequality. This principle result in a partial ordering of populations, as not all populations are directly comparable under this criterion. The partial order forms a lattice and can be equivalently expressed in several ways: sequences of (Pigou-Dalton) transfers, non-intersecting Lorenz curves, stochastic orders, or sums of a convex/concave function [6, 19].

In this section, we first discuss the desired properties of inequality measures relevant to our work and introduce established measures of inequality. We then represent populations using stochastic matrices and depict Lorenz curves as zonogons (convex polygons), since it will simplify the analysis and decomposition properties. Finally, we discuss their operational interpretations and summarize the relationship between population orderings and inequality measures. For a comprehensive resource on fundamental principles and the measurement of inequality, we refer readers to Andreoli and Zoli [20].

#### Inequality metric properties

An inequality measure should satisfy the following five properties:

**Property M1** (Label invariance [9]). *Inequality is invariant to the label of groups or individuals*.

**Property M2** (Duplication invariance [5]).

*Inequality is invariant when duplicating each individual in the population (size invariance)*.

$$\begin{array}{c} I(S)=I(S⊎S) \end{array}$$ (5)

**Property M3** (Scale invariance [21])

*Inequality is invariant under linear scaling of the indicator variable by a factor* $k\in R_{>0}$ *(unit invariance)*.

$$\begin{array}{c} I(S)=I(\{k\cdot s\;:\;s\in S\}) \end{array}$$ (6)

**Property M4** (Pigou-Dalton transfer principle [5, 18]).

*Consider a population* **S** = **G** ⊎ {*s*_1_, *s*_2_} *and a population* $S^{\prime }=G⊎\left\{s_{1}^{\prime },s_{2}^{\prime }\right\}$, *where s*_1_ ≠ *s*_2_ *and* $\left(s_{1}^{\prime },s_{2}^{\prime }\right)$ *is a convex combination of* (*s*_1_, *s*_2_) *with q* ∈ (0, 0.5] *as shown in* Eq (7). *We say* **S**′ *represents the population* **S** *after a Pigou-Dalton transfer between s*_1_ *and s*_2_.

$$\begin{array}{c} \begin{array}{cc} s_{1}^{\prime } & =\left(1-q\right)\cdot s_{1}+q\cdot s_{2}\\ s_{2}^{\prime } & =q\cdot s_{1}+\left(1-q\right)\cdot s_{2} \end{array} \end{array}$$ (7)

- *weak version: A non-zero Pigou-Dalton transfer* (*q* ∈ (0, 0.5]) *can only reduce inequality I*(*S*) ≥ *I*(*S*′).
- *strict version: A non-zero Pigou-Dalton transfer* (*q* ∈ (0, 0.5]) *must reduce inequality I*(*S*) > *I*(*S*′).

*Note that satisfying Property M1 directly extends the range of q* ∈ (0, 0.5] *to q* ∈ (0, 1), *since q* > 0.5 *equals a transfer with relabeling*.

**Property M5** (Non-Negativity with zero at uniform distribution [11]).

- *Inequality is non-negative: I*(**S**) ≥ 0.
- *Inequality is zero if all individuals have an identical indicator value*.
  $$\begin{array}{c} \begin{array}{cc} \left(\forall s_{1},s_{2}\in S\;:\;s_{1}=s_{2}\right) & \Rightarrow I(S)=0 \end{array} \end{array}$$ (8)

**Definition 2**.

*An inequality measure satisfies the ‘weak Property M1-M5’ when considering the weak version of Property M4*.

*An inequality measure satisfies the ‘strict Property M1-M5’ when considering the strict version of Property M4*.

#### Measures of inequality

Several inequality measures are known to satisfy the weak or strict Property M1-M5. The following measures are commonly used in the literature [9]:

- Gini coefficient [22]:
  $$\begin{array}{c} G\left(S\right)=\frac{1}{2\bar{S}{\left|S\right|}^{2}}\sum_{s_{1}\in S}\sum_{s_{2}\in S}\left|s_{1}-s_{2}\right| \end{array}$$ (9)
- Pietra index [23], also known as Ricci-, Schutz- or Hoover index:
  $$\begin{array}{c} R\left(S\right)=\frac{1}{2|S|}\sum_{s\in S}\frac{|s-\bar{S}|}{\bar{S}} \end{array}$$ (10)
- Generalized Entropy index [2]: a parameterized family, the special case of *c* = 1 is known as Theil index [1]. The parameter range (*c*) varies with restrictions on the indicator value.
  $$\begin{array}{c} \begin{array}{cccc} & c\in R\backslash \{0,1\} & \Rightarrow \; & \;{\text{GE}}_{c}\left(S\right)=\frac{1}{c(c-1)}\frac{1}{\left|S\right|}\sum_{s\in S}({\left(\frac{s}{\bar{S}}\right)}^{c}-1)\\ & c=1 & \Rightarrow \; & \;{\text{GE}}_{1}\left(S\right)=\frac{1}{\left|S\right|}\sum_{s\in S}\frac{s}{\bar{S}}\;\text{ln}(\frac{s}{\bar{S}})\\ & c=0 & \Rightarrow \; & \;{\text{GE}}_{0}\left(S\right)=-\frac{1}{\left|S\right|}\sum_{s\in S}\text{ln}(\frac{s}{\bar{S}}) \end{array} \end{array}$$ (11)
- Atkinson indexes [6]: a parameterized family, designed with the properties of a welfare function in mind. It can be represented as transformation of the Generalized entropy index [24].
  $$\begin{array}{c} \begin{array}{cccccc} & d\in (0,1) & \Rightarrow \; & \;A_{d}\left(S\right)=1-{\left[d\left(d-1\right){\text{GE}}_{1-d}\left(S\right)+1\right]}^{\frac{1}{1-d}} & & =1-\frac{1}{\bar{S}}{\left(\frac{1}{\left|S\right|}\sum_{s\in S}s^{1-d}\right)}^{\frac{1}{1-d}}\\ & d=1 & \Rightarrow \; & \;A_{1}\left(S\right)=1-e^{-{\text{GE}}_{0}\left(S\right)} & & =1-\frac{1}{\bar{S}}{\left(\prod_{s\in S}s\right)}^{\frac{1}{\left|S\right|}} \end{array} \end{array}$$ (12)

### Lorenz curves and their ordering

The Lorenz curve represents the minimal concentration of wealth in a subgroup of a particular size and is typically defined through the Quantile function [9, 24]. However, we can equivalently define the Lorenz curve as the boundary of a zonogon [25, 26]. This directly highlights the well-known relation [1, 6] between the Lorenz curve [27], the Neyman-Pearson region from hypothesis testing [28, p. 278], and the (pointwise) Blackwell order from information theory [4, 29]. Moreover, the definition through zonogons highlights additional properties of the inequality measures defined in Section “Methodology”.

#### Zonogons and their partial order

**Definition 3** (Stochastic matrix). *A (row) stochastic matrix* $\lambda \in R_{\geq 0}^{a\times b}$ *of dimension a* × *b is a matrix, where all entries are non-negative real values and each row sums to one. In a double stochastic matrix, all entries are non-negative, and each row and column sums to one*.

**Definition 4** (Normalized population matrix). *We define a function* $\kappa :P_{M}\left(R_{\geq 0}\right)\to R_{\geq 0}^{2\times m}$ *as shown in* Eq (13). *The function maps a population* **S** *to a* 2 × |**S**| *row stochastic matrix by normalizing both, the population size and indicator value. The ordering of columns can be arbitrary (discussed below Definition 5)*.

κS≔1S1sS¯:s∈S (13)

**Example 2**. *A normalized population matrix for* Γ({1, 2}, **M**) *from Example 1 is shown in* Eq (14) *(columns can be permuted)*.

$$\begin{array}{c} \kappa \left(\Gamma \left(\left\{1,2\right\},M\right)\right)=[\begin{array}{cccc} 1/4 & 1/4 & 1/4 & 1/4\\ 1/10 & 4/10 & 4/10 & 1/10 \end{array}] \end{array}$$ (14)

**Notation 2**. *We access a vector within a normalized population matrix as* ${\vec{v}}_{i}\in \kappa \left(S\right)$, *such as* v→i=1S(SiS¯1).

**Definition 5 (Zonogon** [25, 26, 29]). *The function* $Z:R_{\geq 0}^{2\times n}\to P\left({\left[0,1\right]}^{2}\right)$ *transforms a normalized population matrix into a zonogon. A zonogon* (Eq (15)) *is a set of two-dimensional points constructed from the Minkowski sum of line segments from its generating vectors* ${\vec{v}}_{i}\in \kappa \left(S\right)$.

$$\begin{array}{c} Z(\kappa \left(S\right))≔\{\sum_{i=1}^{\left|S\right|}x_{i}{\vec{v}}_{i}\;:\;x_{i}\in \left[0,1\right],\;{\vec{v}}_{i}\in \kappa \left(S\right)\}=\{\kappa \left(S\right)a\;:\;a\in {\left[0,1\right]}^{\left|S\right|}\} \end{array}$$ (15)

The zonogon can be defined equivalently as image of the unit-cube [0, 1] ^|**S**|^ under the linear transformation of the given matrix and provides the following basic properties [29]:

- The zonogon of a stochastic matrix is a centrally symmetric convex polygon.
- The zonogon is invariant to permuting the order of matrix vectors:

$Z([\begin{array}{cccc} {\vec{v}}_{1} & \ldots & {\vec{v}}_{2} & \ldots \end{array}])=Z([\begin{array}{cccc} {\vec{v}}_{2} & \ldots & {\vec{v}}_{1} & \ldots \end{array}])$ .
- The zonogon is invariant to splitting/merging matrix vectors of identical slope:

$Z([\begin{array}{cc} (1+l){\vec{v}}_{1} & \ldots \end{array}])=Z([\begin{array}{ccc} {\vec{v}}_{1} & l{\vec{v}}_{1} & \ldots \end{array}])$ .
- Ordering the matrix vectors ${\vec{v}}_{i}\in \kappa \left(S\right)$ by increasing/decreasing slope provides the zonogon perimeter (visualized in Fig 4A).

**Notation 3**. *For abbreviation, we use the notation Z*_*κ*_(**S**) ≔ *Z*(*κ*(**S**)).

Zonogon examples and their interpretation are discussed in Example 5 of Section “Operational meaning of zonogons”.

**Definition 6** (Zonogon order [29]). *The subset relation* (Eq (16a)) *is a partial order of zonogons from* 2 ×_*row stochastic matrices that forms a (non-distributive) lattice with unique meet and join elements. Under this ordering relation, the meet of two zonogons corresponds to their intersection and their join corresponds to the convex hull of their union*.

$$\begin{array}{c} Z_{\kappa }\left(S_{1}\right)\;\subseteq Z_{\kappa }\left(S_{2}\right)\; \end{array}$$ (16a)

$$\begin{array}{c} \Leftrightarrow \;\kappa (S_{1})\;=\kappa (S_{2})\;\lambda \;for\;some\;row\;stochastic\;matrix\;\lambda \end{array}$$ (16b)

A zonogon is a subset of another *Z*_*κ*_(**S**_1_) ⊆ *Z*_*κ*_(**S**_2_) if and only if there exists a row stochastic matrix λ such that *κ*(**S**_1_) = *κ*(**S**_2_)λ (Eq (16b)) [29]. This relation leads to Eq (17), which is useful since any sequence of Pigou-Dalton transfers corresponds to a multiplication by some stochastic matrix (see S1 Appendix).

$$\begin{array}{c} Z(\kappa (S)\;\lambda )\subseteq Z(\kappa (S))\;\text{for}\;\text{all}\;\text{row}\;\text{stochastic}\;\text{matrices}\;\lambda \end{array}$$ (17)

We can use the lattice of zonogons to define a lattice of population equivalence classes.

**Definition 7** (Population equivalence). *We say two populations* (**S**_1_, **S**_2_) *are equivalent (≅) if and only if they generate the same zonogon*.

$$\begin{array}{c} (S_{1}≅S_{2})≔\left(Z_{\kappa }\left(S_{1}\right)=Z_{\kappa }\left(S_{2}\right)\right) \end{array}$$ (18)

**Notation 4**.

- *We notate the equivalence class of a population as* $\langle S_{1}\rangle ≔\left\{S_{2}\in P\left(P_{M}\left(R_{\geq 0}\right)\right)\;:\;S_{2}≅S_{1}\right\}$ .
- *We extend the notation for zonogons to equivalence classes Z*_*κ*_(〈**S**〉) ≔ *Z*_*κ*_(**S**).

**Definition 8** (Lattice of population equivalence classes). *The lattice of zonogons provides a lattice for the equivalence classes of populations. We notate their ordering as* 〈**S**_1_〉 ⊑ 〈**S**_2_〉, *their meet as* 〈**S**_1_〉 ⊓ 〈**S**_2_〉 *and join as* 〈**S**_1_〉 ⊔ 〈**S**_2_〉. *We notate a top and bottom population for the lattice as* ⊤_***S***_ = {0, 1} *and* ⊥_***S***_ = {1} *respectively*. Conv(·) *indicates the convex hull in* Eq (19d).

$$\begin{array}{c} (\left\langle S_{1}\right\rangle ⊑\left\langle S_{2}\right\rangle )≔\left(Z_{\kappa }\left(S_{1}\right)\subseteq Z_{\kappa }\left(S_{2}\right)\right) \end{array}$$ (19a)

$$\begin{array}{c} (\left\langle S_{1}\right\rangle \subset \left\langle S_{2}\right\rangle )≔\left(Z_{\kappa }\left(S_{1}\right)\subset Z_{\kappa }\left(S_{2}\right)\right) \end{array}$$ (19b)

$$\begin{array}{c} Z_{\kappa }\left(\left\langle S_{1}\right\rangle ⊓\left\langle S_{2}\right\rangle \right)≔=Z_{\kappa }\left(S_{1}\right)\cap Z_{\kappa }\left(S_{2}\right) \end{array}$$ (19c)

$$\begin{array}{c} Z_{\kappa }\left(\left\langle S_{1}\right\rangle ⊔\left\langle S_{2}\right\rangle \right)=\text{Conv}(Z_{\kappa }\left(S_{1}\right)\cup Z_{\kappa }\left(S_{2}\right)) \end{array}$$ (19d)

**Notation 5**. *The equivalence class of the ‘joint’ distribution for two attributes is* 〈Γ({1, 2}, **M**)〉, *while the ‘join’ of both attributes is* 〈Γ({1}, **M**)〉 ⊔ 〈Γ({2}, **M**)〉.

To obtain a set-theoretic behavior of inequality measures, we have to understand the inclusion-exclusion relation between the defined lattice operations. For an example of this concept, we can first use the standard set-theoretic inclusion-exclusion relation (|*A* ∪ *B*| = |*A*| + |*B*| − |*A* ∩ *B*|) to obtain Eq (20a) where the cardinality of the set is expressed as area of the zonogon: For a non-empty set of populations (∅ ≠ **A**), computing an inclusion-exclusion principle on the zonogon area of the meet (zonogon intersection) gives the area of their union, which is a lower bound on the area of their join (convex hull of the union). We can separate terms based on their sign (Eq (20b)) to recognize another inclusion-exclusion principle later (Eq (23)).

$$\begin{array}{c} \text{Area}(Z_{\kappa }(\underset{S\in A}{⊔}\left\langle S\right\rangle ))\geq \text{Area}(\underset{S\in A}{⊔}Z_{\kappa }\left(S\right))=\sum_{\emptyset \neq B\subseteq A}{\left(-1\right)}^{|B|-1}\text{Area}(Z_{\kappa }(\underset{S\in B}{⊓}\left\langle S\right\rangle )) \end{array}$$ (20a)

$$\begin{array}{c} \text{Area}(Z_{\kappa }(\underset{S\in A}{⊔}\left\langle S\right\rangle ))+\sum_{\overset{\emptyset \neq B\subseteq A}{|B|\;\text{even}}}\text{Area}(Z_{\kappa }(\underset{S\in B}{⊓}\left\langle S\right\rangle ))\geq \sum_{\overset{B\subseteq A}{|B|\;\text{odd}}}\text{Area}(Z_{\kappa }(\underset{S\in B}{⊓}\left\langle S\right\rangle )) \end{array}$$ (20b)

**Example 3**. *Consider two populations* **A** = {**S**_1_, **S**_2_}. *The classical inclusion-exclusion relation of* Eq (20) *becomes* Eq (21).

$$\begin{array}{c} \text{Area}(Z_{\kappa }(\left\langle S_{1}\right\rangle ⊔\left\langle S_{2}\right\rangle ))\;\geq \;\text{Area}(Z_{\kappa }(\langle S_{1}\rangle ))+\text{Area}(Z_{\kappa }(\langle S_{2}\rangle ))-\text{Area}(Z_{\kappa }(\left\langle S_{1}\right\rangle ⊓\left\langle S_{2}\right\rangle )) \end{array}$$ (21)

**Fig 2:**
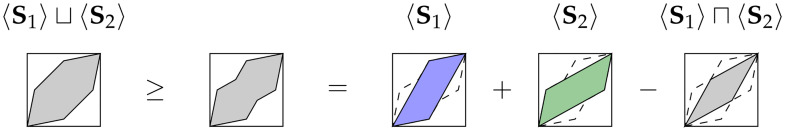
Visualization of Eqs (20) and (21). The inclusion-exclusion relation provides the set-theoretic zonogon union (ride-hand side). The area of the set-theoretic union is always a subset of their convex hull (left-hand side) and thus has a smaller area.

*This relation is visualized in* Fig 2: *The left-hand side represents the convex hull of both zonogons, while the ride-hand side represents their set-theoretic union*.

*From the perspective of* Eq (20), *the inclusion-exclusion relation on the meet (⊓) provides a lower bound for the join* (⊔).

Measuring the zonogon area is well known as Gini coefficient [1, p. 121], which provides a clear inclusion-exclusion relation (Area inclusion-exclusion). However, we will define another family of inequality measures in Section “Defining f-inequality”, such that the inclusion-exclusion relation relates to the Minkowski sum of zonogons (Definition 9). This family of inequality measures generalizes important inequality measures and will provide their decomposition (e.g. Pietra index, Generalized Entropy index, Atkinson index). Therefore, we discuss the inclusion-exclusion relation of zonogons at the Minkowski sum next:

**Definition 9** (Zonogon sum). *The addition of two zonogons corresponds to their Minkowski sum:*

$$\begin{array}{c} Z_{\kappa }\left(\left\langle S_{1}\right\rangle \right)+Z_{\kappa }\left(\left\langle S_{2}\right\rangle \right)≔Z_{\kappa }\left(S_{1}\right)+Z_{\kappa }\left(S_{2}\right)≔\;\{a+b\;:\;a\in Z_{\kappa }\left(S_{1}\right),\;b\in Z_{\kappa }\left(S_{2}\right)\} \end{array}$$ (22a)

$$\begin{array}{cc} \; & \;=Z([\begin{array}{cc} \kappa (S_{1}) & \kappa (S_{2}) \end{array}]) \end{array}$$ (22b)

The defined operators provide the following inclusion-exclusion relation at the zonogon sum [4, Lemma A^5^].

$$\begin{array}{c} Z_{\kappa }(\underset{S\in A}{⊓}\left\langle S\right\rangle )+\sum_{\overset{\emptyset \neq B\subseteq A}{|B|\;\text{even}}}Z_{\kappa }(\underset{S\in B}{⊔}\left\langle S\right\rangle )\subseteq \sum_{\overset{B\subseteq A}{|B|\;\text{odd}}}Z_{\kappa }(\underset{S\in B}{⊔}\left\langle S\right\rangle ) \end{array}$$ (23)

**Fig 3:**
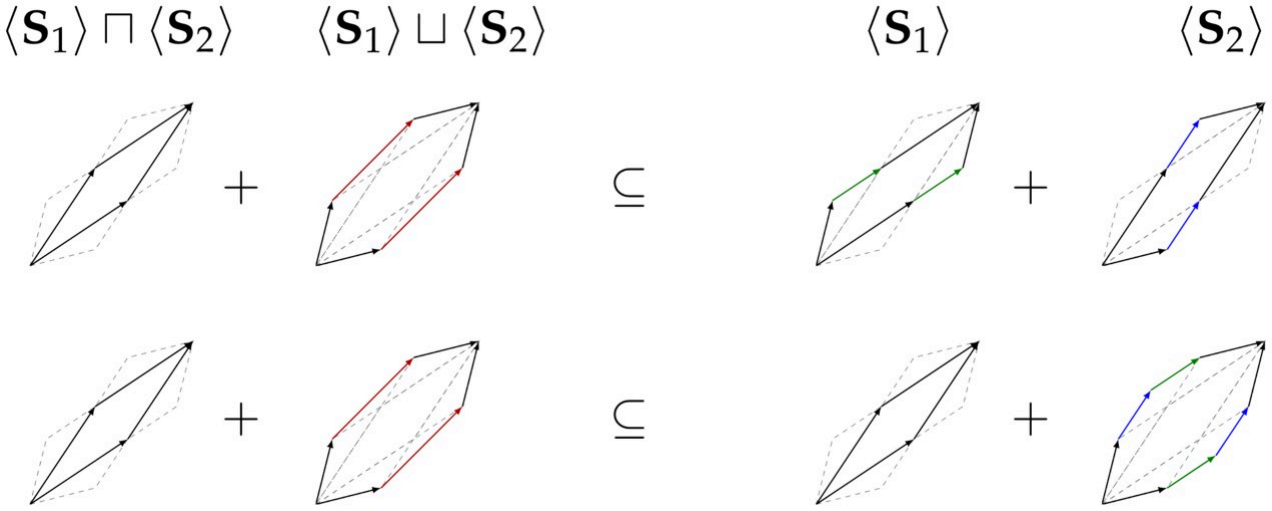
Visualization of Eq (23) and Eq (24). The zonogon sum equals concatenating the generating vectors as highlighted in Eq (22b). The zonogon sum for **S**_1_ and **S**_2_ contains the vectors forming their intersection and union. However, the vectors forming the union are re-ordered to maintain the convexity of a zonogon. The result is a superset of the convex hull (join).

**Example 4**. *Consider two populations* **A** = {**S**_1_, **S**_2_}. *The inclusion-exclusion relation of* Eq (23) *becomes* Eq (24) *and is visualized in* Fig 3. *The plus operation corresponds to the Minkowski sum (Definition 9)*.

$$\begin{array}{c} Z_{\kappa }(\left\langle S_{1}\right\rangle ⊓\left\langle S_{2}\right\rangle )+Z_{\kappa }(\left\langle S_{1}\right\rangle ⊔\left\langle S_{2}\right\rangle )\;\subseteq \;Z_{\kappa }(\langle S_{1}\rangle )+Z_{\kappa }(\langle S_{2}\rangle ) \end{array}$$ (24)

*From the perspective of* Eq (23), *the inclusion-exclusion relation on the join (*⊔*) provides an upper bound for the meet (*⊓).

#### Operational meaning of zonogons

**Fig 4:**
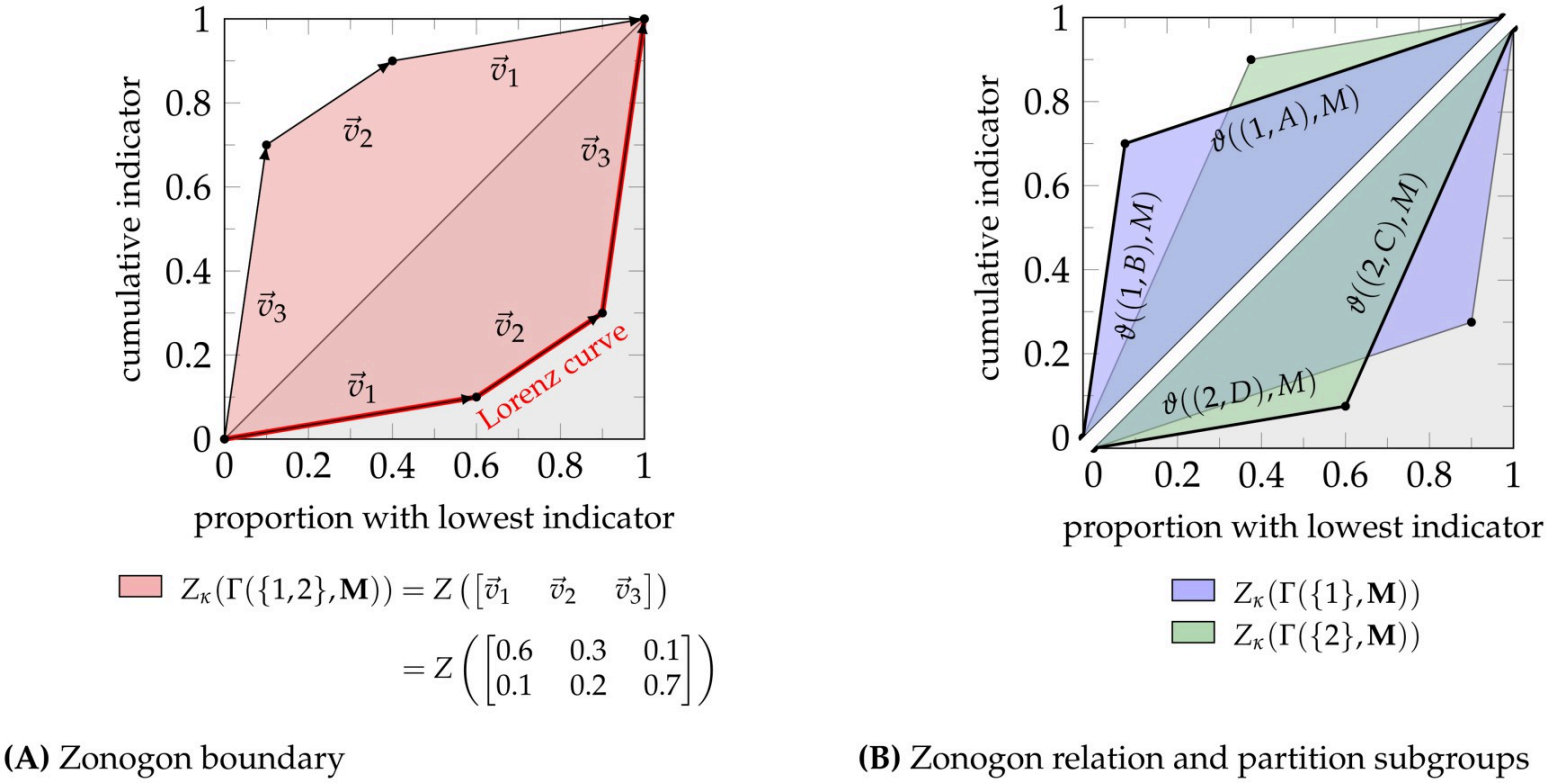
Zonogon construction, the meaning of its boundary and their ordering. (A) The zonogon of a population is a symmetric convex polygon containing the line from (0,0) to (1,1). Sorting the vectors of a normalized population matrix by increasing slope provides the lower boundary of the zonogon, which is the Lorenz curve. (B) Each zonogon boundary segment corresponds to one subgroup of the partition, and its slope is the expected normalized indicator value of its individuals. The subgroups for the partition on attribute $A_{1}$ are labeled in the upper triangle and those for the partition on $A_{2}$ are labeled in the lower triangle. The example was constructed such that the join of both attributes (Fig 4B) equals their joint distribution (Fig 4A). For any other attribute dependence, the zonogon of their joint distribution is a superset of Fig 4A.

**Definition 10** (Lorenz Curve [27]). *The Lorenz curve maps a fraction of the population (x-axis) to the minimal fraction of the indicator value (y-axis) concentrated in any subgroup of this size. The Lorenz curve is the lower boundary of the zonogon (Definition 5, visualized in* Fig 4A) [25, 26].

**Remark 2**. *For all practical purposes, we encourage the reader to view zonogons as Lorenz curves*.

**Definition 11** (Atkinson criterion). *Assume two populations* (**S**_1_, **S**_2_) *with identical indicator mean* (${\bar{S}}_{1}={\bar{S}}_{2}$) *and let the welfare of a population be the expected value of an increasing concave function w*(⋅). *Some populations can be compared without agreeing on the specific function w*(⋅), *which leads to the Atkinson criterion shown in* Eq (25a) [6].

$$\begin{array}{cccc} & \forall w\;:\;\frac{1}{|S_{2}|}\sum_{s\in S_{2}}w\left(s\right)\leq \frac{1}{|S_{1}|}\sum_{s\in S_{1}}w\left(s\right) & & where\;w(\cdot )\;is\;increasing\;and\;concave \end{array}$$ (25a)

$$\begin{array}{cccc} \Leftrightarrow \; & \forall v\;:\;\frac{1}{|S_{1}|}\sum_{s\in S_{1}}v\left(s\right)\leq \frac{1}{|S_{2}|}\sum_{s\in S_{2}}v\left(s\right) & & where\;v(t)≔-w(t)\;is\;decreasing\;and\;convex \end{array}$$ (25b)

$$\begin{array}{cccc} \Leftrightarrow \; & \exists \lambda \;:\;\kappa \left(S_{1}\right)=\kappa \left(S_{2}\right)\;\lambda & & where\;\lambda \;is\;a\;row\;stochastic\;matrix \end{array}$$ (25c)

$$\begin{array}{cc} \Leftrightarrow \; & Z_{\kappa }\left(S_{1}\right)\subseteq Z_{\kappa }\left(S_{2}\right) \end{array}$$ (25d)

$$\begin{array}{cc} \Leftrightarrow \; & \;\;\left\langle S_{1}\right\rangle ⊑\left\langle S_{2}\right\rangle \end{array}$$ (25e)

For the context of this work, we can change the perspective from higher welfare to lower inequality (Eq (25b)), where the convexity of *v*(⋅) leads to Jensen’s inequality. Atkinson [6] showed that a population has a higher welfare (in this context: lower inequality) for any *w* (in this context: *v*) if and only if there exists a sequence of Pigou-Dalton transfers from **S**_2_ to **S**_1_ (Eq (25c)). This equals the condition of non-intersecting Lorenz curves and the zonogon order (Eq (25d)) [6].

**Remark 3**. *The condition of an identical indicator mean in Definition 11 has no further importance if the inequality measure is invariant to the population size and scaling of the indicator variable (Property M1-M3). In this case, normalizing the population size and indicator variable always results in the same mean without affecting the inequality measure*.

**Example 5**. *Consider the model **M** obtained from* Table 1 *with the two attributes* $A_{1}=\left\{A,B\right\}$ *and* $A_{2}=\left\{C,D\right\}$.

**Table 1:** Example population model.

Indicator value	$A_{1}$	$A_{2}$	Number of individuals
16	A	D	6
23	A	C	3
7	B	C	1
Total sum: 10	/	/	10

*We can construct the following three partitions based on the given attributes. To abbreviate the notation, we can sum columns with an identical slope without affecting the underlying zonogon, as discussed above. The order of columns is arbitrary*.

ZκΓ1,2,M=Z0.10.10.10.10.10.10.10.10.10.1160160160160160160230230230730 (26a)

$$\begin{array}{cc} & =Z([\begin{array}{ccc} 0.6 & 0.3 & 0.1\\ 0.1 & 0.2 & 0.7 \end{array}]) \end{array}$$ (26b)

ZκΓ1,M=Z0.10.10.10.10.10.10.10.10.10.1710130130130130130130130130130 (26c)

$$\begin{array}{c} =Z([\begin{array}{cc} 0.1 & 0.9\\ 0.7 & 0.3 \end{array}]) \end{array}$$ (26d)

ZκΓ2,M=Z0.10.10.10.10.10.10.10.10.10.1160160160160160160940940940940 (26e)

$$\begin{array}{cc} & =Z([\begin{array}{cc} 0.6 & 0.4\\ 0.1 & 0.9 \end{array}]) \end{array}$$ (26f)

*The zonogon for each partition is shown in* Fig 4. *The lower boundary of the zonogon is the Lorenz curve* (Fig 4A). *Each edge segment of the zonogon corresponds to one subgroup of the partition, and its slope represents the expected normalized indicator value of the individuals within it* (Fig 4B). *As shown in* Fig 4B, *the partitions* Γ({1}, **M**) *and* Γ({2}, **M**) *are incomparable since neither zonogon is a subset of the other. This means that there does not exist a sequence of Pigou-Dalton transfers to convert one population into the other and that the preferable partition depends on the considered inequality measure. However, we can always construct Pigou-Dalton transfers to eliminate an attribute, which leads to the relation of* Eq (27).

$$\begin{array}{c} a\subseteq b\Rightarrow Z_{\kappa }\left(\Gamma \left(a,M\right)\right)\subseteq Z_{\kappa }\left(\Gamma \left(b,M\right)\right) \end{array}$$ (27)

*The numbers of this particular example* (Table 1) *were chosen such that the joint attribute distribution corresponds to the join of partitioning on the individual attributes:* 〈Γ({1, 2}, **M**)〉 = 〈Γ({1}, **M**)〉 ⊔ 〈Γ({2}, **M**)〉. *The join plays an important role since it represents the dependence between attributes* ($A_{1}$, $A_{2}$) *that leads to a zonogon that is unique and a subset of any other dependence. Thus, we can construct Pigou-Dalton transfers from all other attribute dependencies to arrive at the join population. The attribute dependence of the join provides minimal inequality under any measure satisfying Property M1-M5. Therefore, it represents a notion of ideal attribute dependence and demonstrates that the ideal dependence between attributes is measure independent*.

#### From ordering to quantification

We can simplify the required properties of inequality measures for the remaining context of this work by using the ordering of population equivalence classes. While the relation between population orderings and inequality properties is well established [20], we distinguish two cases that will be used to construct inequality measures in the following section:

**Property (weak ⋆)**. *The inequality measure I*(⋅) *shall maintain the zonogon order and quantify a bottom population* (⊥_**S**_) *to zero* (Eq (28)).

$$\begin{array}{c} \left\langle S_{1}\right\rangle ⊑\left\langle S_{2}\right\rangle \Rightarrow I\left(S_{1}\right)\leq I\left(S_{2}\right), \end{array}$$ (28a)

$$\begin{array}{cc} I({⊥}_{S}) & =0. \end{array}$$ (28b)

**Property (strict ⋆)**. *The inequality measure I*(⋅) *shall maintain the strict zonogon order and quantify a bottom population* (⊥_**S**_) *to zero* (Eq (29)).

$$\begin{array}{c} \left\langle S_{1}\right\rangle =\left\langle S_{2}\right\rangle \Rightarrow I\left(S_{1}\right)=I\left(S_{2}\right), \end{array}$$ (29a)

$$\begin{array}{c} \left\langle S_{1}\right\rangle \subset \left\langle S_{2}\right\rangle \Rightarrow I\left(S_{1}\right)<I\left(S_{2}\right), \end{array}$$ (29b)

$$\begin{array}{cc} I({⊥}_{S}) & =0. \end{array}$$ (29c)

**Lemma 1**. *Satisfying Property* (*weak* ⋆) *implies that the inequality measure satisfies the weak Property M1-M5*.

**Lemma 2**. *Satisfying Property* (*strick* ⋆) *implies that the inequality measure satisfies the strict Property M1-M5*.

The proof of Lemma 1 and 2 is shown in S1 Appendix. The relation between some inequality measures and the Lorenz curve (and thus their zonogons) is well established: The Gini coefficient is known to equal twice the area between the Lorenz curve and diagonal [1, p. 121]. Thus, the Gini coefficient equals the zonogon area (*G*(**S**) = Area *Z*_*κ*_(**S**)) and satisfies Property (strick ⋆). The Pietra index is known to equal the maximal vertical distance between the Lorenz curve and diagonal [9, p. 17] and thus satisfies Property (weak ⋆).

## Methodology

We begin by defining a family of inequality measures (Section “Defining f-inequality”) and demonstrate that several established measures are its special case. Section “Preliminary intuition for redundancy and synergy” provides an intuition for the concepts of redundancy and synergy and highlights the limitation of analyses with Shapley values in this setting. With this motivation, we explain the decomposition lattice and desired properties for a set-theoretic intuition (Section “Decomposition lattice and required properties”). Section “Decomposing f-inequality” defines a decomposition that satisfies the desired properties and provides a suitable operational interpretation. We demonstrate how the decomposition results can be transformed to other inequality measures, such as an Atkinson index (Section “Decomposing the Atkinson index”). Finally, Section “Multi-layered inequality” discusses multi-layered inequality and Section quotes The relation of inequality and information highlights the relation between decomposing measures of information and inequality.

### Defining f-inequality

If a zonogon is a subset of another, then it shall obtain a smaller inequality score to obtain Property M1-M5 from Lemma 1 and 2. For a first intuition, consider quantifying the length of the zonogon boundary (Lorenz curve): All zonogons are convex and have a common start and end point. Therefore, if a zonogon is a subset of another (Atkinson criterion), then its boundary is shorter.

For turning this conceptual idea into a family of inequality measures, we can follow a simple strategy: (1) Define the inequality measure as sum of quantifying each vector in the normalized population matrix (zonogon boundary segment) by a function *r*. This could be re-phrased to a sum of quantifying each individual of the population. (2) The function *r* shall satisfy three properties: (a) quantify any vector of slope one to zero, (b) scale linearly, and (c) be convex. Quantifying any vector of slope one to a score of zero ensures quantifying the bottom element (⊥_**S**_) correctly. The linear scaling and convexity provide a triangle inequality on the zonogon boundary, which then reflects their subset relation on the inequality measure. Interestingly, we previously studied a function that satisfies exactly these properties for decomposing information measures [4].

**Notation 6**. *We reserve the name f for generator functions of an f*-*divergence* [30]: *Let* f:(0,∞)→R *be a function that satisfies the following three properties. By convention we understand that* $f(0)={\text{lim}}_{t\to 0^{+}}f(t)$ *and* $0f(\frac{0}{0})=0$:

- *f is convex*,
- *f*(1) = 0,
- *f*(*t*) *is finite for all t* > 0.

**Definition 12** (*f*-inequality).

- *Define a function r*_*f*,*p*_ *as shown in* Eq (30a) *to quantify a vector* $\vec{v}={[}_{y}^{x}]$ *of the zonogon boundary with p*, *x*, *y* ∈ [0, 1].
- *Define a parameterized class of f*-*inequality measures* (*p* ∈ [0, 1]) *as shown in* Eq (30b) *to be the sum of all segments from the Lorenz curve for a populations* **S**.

$$\begin{array}{c} r_{f,p}({[}_{y}^{x}])≔\left(px+(1-p)y)\cdot f(\frac{x}{px+(1-p)y}\right) \end{array}$$ (30a)

$$\begin{array}{c} I_{f,p}(S)≔\sum_{\vec{v}\in \kappa \left(S\right)}r_{f,p}\left(\vec{v}\right) \end{array}$$ (30b)

$$\begin{array}{c} =\frac{1}{\left|S\right|}\sum_{s\in S}\frac{f(g(p,\bar{S},s))}{g(p,\bar{S},s)}\;where:\;\;\;g\left(p,\bar{S},s\right)=\frac{\bar{S}}{p\bar{S}+\left(1-p\right)s} \end{array}$$ (30c)

**Notation 7**.

*We say an f*-*inequality measure is ‘strict’ if and only if its generator function f is strictly convex*.

*We say an f-inequality measure is ‘weak’ if and only if its generator function f is not strictly convex*.

**Theorem 1** (Properties of *r*_*f*,*p*_ and *I*_*f*,*p*_) *For a constant p* ∈ [0, 1]:

*1. the function* $r_{f,p}\left(\vec{v}\right)$ :
  *(a) quantifies any vector of slope one to zero:* $r_{f,p}({[}_{\ell }^{\ell }])=0$
  *(b) quantifies the zero vector to zero:* $r_{f,p}({[}_{0}^{0}])=0$

  *(c) scales linearly in* $\vec{v}$ *where* ℓ∈R: $r_{f,p}\left(\ell \vec{v}\right)=\ell r_{f,p}\left(\vec{v}\right)$

  *(d) is convex in* $\vec{v}$ :
    - *f-inequality ℓ* ∈ {0, 1}:

$r_{f,p}\left(\ell {\vec{v}}_{1}+\left(1-\ell \right){\vec{v}}_{2}\right)=\ell r_{f,p}\left({\vec{v}}_{1}\right)+\left(1-\ell \right)r_{f,p}\left({\vec{v}}_{2}\right)$

    - *weak f-inequality ℓ* ∈ (0, 1):

$r_{f,p}\left(\ell {\vec{v}}_{1}+\left(1-\ell \right){\vec{v}}_{2}\right)\leq \ell r_{f,p}\left({\vec{v}}_{1}\right)+\left(1-\ell \right)r_{f,p}\left({\vec{v}}_{2}\right)$

    - *strict f-inequality ℓ* ∈ (0, 1):

$r_{f,p}\left(\ell {\vec{v}}_{1}+\left(1-\ell \right){\vec{v}}_{2}\right)<\ell r_{f,p}\left({\vec{v}}_{1}\right)+\left(1-\ell \right)r_{f,p}\left({\vec{v}}_{2}\right)$

  *(e) satisfies a triangle inequality in* $\vec{v}$ :
    - *f-inequality* $\text{Slope}\left({\vec{v}}_{1}\right)=\text{Slope}\left({\vec{v}}_{2}\right)$ :

$r_{f,p}\left({\vec{v}}_{1}+{\vec{v}}_{2}\right)=r_{f,p}\left({\vec{v}}_{1}\right)+r_{f,p}\left({\vec{v}}_{2}\right)$

    - *weak f-inequality* $\text{Slope}\left({\vec{v}}_{1}\right)\neq \text{Slope}\left({\vec{v}}_{2}\right)$ :

$r_{f,p}\left({\vec{v}}_{1}+{\vec{v}}_{2}\right)\leq r_{f,p}\left({\vec{v}}_{1}\right)+r_{f,p}\left({\vec{v}}_{2}\right)$

    - *strict f-inequality* $\text{Slope}\left({\vec{v}}_{1}\right)\neq \text{Slope}\left({\vec{v}}_{2}\right)$ :

$r_{f,p}\left({\vec{v}}_{1}+{\vec{v}}_{2}\right)<r_{f,p}\left({\vec{v}}_{1}\right)+r_{f,p}\left({\vec{v}}_{2}\right)$

*2. the function I*_*f*,*p*_(**S**):
  *(a) quantifies the bottom element to zero:*     *I*_*f*,*p*_(⊥_**S**_) = 0
*(b) maintains the zonogon order:*
    - *f-inequality:*     〈**S**_1_〉 = 〈**S**_2_〉 ⇒ *I*_*f*,*p*_(**S**_1_) = *I*_*f*,*p*_(**S**_2_)
    - *weak f-inequality:*     〈**S**_1_ 〉⊑ 〈**S**_2_〉 ⇒ *I*_*f*,*p*_(**S**_1_) ≤ *I*_*f*,*p*_(**S**_2_)
    - *strict f-inequality:*     〈**S**_1_〉 ⊏ 〈**S**_2_〉 ⇒ *I*_*f*,*p*_(**S**_1_) < *I*_*f*,*p*_(**S**_2_)

The proof of Theorem 1 is shown in S2 Appendix.

**Corollary 1**.

- *Any weak f-inequality satisfies Property (weak ⋆) and the weak Property M1-M5*.
- *Any strict f-inequality satisfies Property (strick ⋆) and the strict Property M1-M5*.

*Proof*. Follows directly from Theorem 1 with Lemma 1 and Lemma 2.

**Notation 8**. *Since f-inequality is equal for all populations within an equivalence class (Theorem 1 nr. 2b), we can quantify an equivalence class by any population that it contains:* *I*_*f*,*p*_(〈**S**〉) ≔ *I*_*f*,*p*_(**S**).

The intended attribute decomposition will require an interpretation for the addition of inequality from multiple populations. Therefore, it will be helpful that the Minkowski sum of the underlying zonogons directly corresponds to the addition of *f*-inequality from their generating populations.

**Lemma 3**. *Consider two non-empty sets of populations with equal cardinality* (|**A**| = |**B**|), *then*:

$$\begin{array}{ccc} f\text{-}inequality: & & \sum_{S\in A}Z_{\kappa }\left(S\right)=\sum_{S\in B}Z_{\kappa }\left(S\right)\Rightarrow \sum_{S\in A}I_{f,p}\left(S\right)=\sum_{S\in B}I_{f,p}\left(S\right) \end{array}$$ (31a)

$$\begin{array}{ccc} weak\;f\text{-}inequality: & & \sum_{S\in A}Z_{\kappa }\left(S\right)\subseteq \sum_{S\in B}Z_{\kappa }\left(S\right)\Rightarrow \sum_{S\in A}I_{f,p}\left(S\right)\leq \sum_{S\in B}I_{f,p}\left(S\right) \end{array}$$ (31b)

$$\begin{array}{ccc} strict\;f\text{-}inequality: & & \sum_{S\in A}Z_{\kappa }\left(S\right)\subset \sum_{S\in B}Z_{\kappa }\left(S\right)\Rightarrow \sum_{S\in A}I_{f,p}\left(S\right)<\sum_{S\in B}I_{f,p}\left(S\right) \end{array}$$ (31c)

The proof of Lemma 3 is shown in S2 Appendix.

**Corollary 2**. *Any f-inequality satisfies the following inclusion-exclusion relation:*

$$\begin{array}{c} \begin{array}{cc} I_{f,p}(\underset{S\in A}{⊓}\left\langle S\right\rangle ) & \leq \sum_{\emptyset \neq B\subseteq A}{\left(-1\right)}^{|B|-1}I_{f,p}(\underset{S\in B}{⊔}\left\langle S\right\rangle ) \end{array} \end{array}$$ (32)

*Proof*. Follows directly from Lemma 3 and Eq (23).

**Theorem 2**. *The Pietra index and Generalized Entropy index are special cases of f-inequality:*

$$\begin{array}{ccc} R(S)=I_{f,p}\left(S\right) & where:\; & p=0\;\;and\;\;f\left(t\right)=\frac{\left|t-1\right|}{2} \end{array}$$ (33a)

$$\begin{array}{ccc} {GE}_{c}\left(S\right)=I_{f,p}\left(S\right) & where:\; & p=0\;\;and\;\;f\left(t\right)=\frac{t^{1-c}-t}{c(c-1)} \end{array}$$ (33b)

$$\begin{array}{ccc} {GE}_{1}\left(S\right)=I_{f,p}\left(S\right) & where:\; & p=0\;\;and\;\;f\left(t\right)=-\text{ln}(t) \end{array}$$ (33c)

$$\begin{array}{ccc} {GE}_{0}\left(S\right)=I_{f,p}\left(S\right) & where:\; & p=0\;\;and\;\;f\left(t\right)=t\;\text{ln}(t) \end{array}$$ (33d)

The proof of Theorem 2 is shown separately in S2 Appendix. This section presented the construction of inequality measures from any *f*-divergence.

Finally, we discuss the impact of the introduced parameter *p*: As it can be seen from Eq (30c), this parameter is equivalent to a pre-processing that does not affect the average indicator value of the population ($\bar{S}$) but shifts all individuals closer to the average $s_{new}=p\bar{S}+\left(1-p\right)s_{old}$. Therefore, increasing *p* reduces the overall inequality until it reaches zero at *p* = 1. However, when viewing each parameter *p* as generating its own inequality measure, then we can find that changing *p* changes how the measure ranks Lorenz incomparable populations as demonstrated in Example 6.

**Fig 5:**
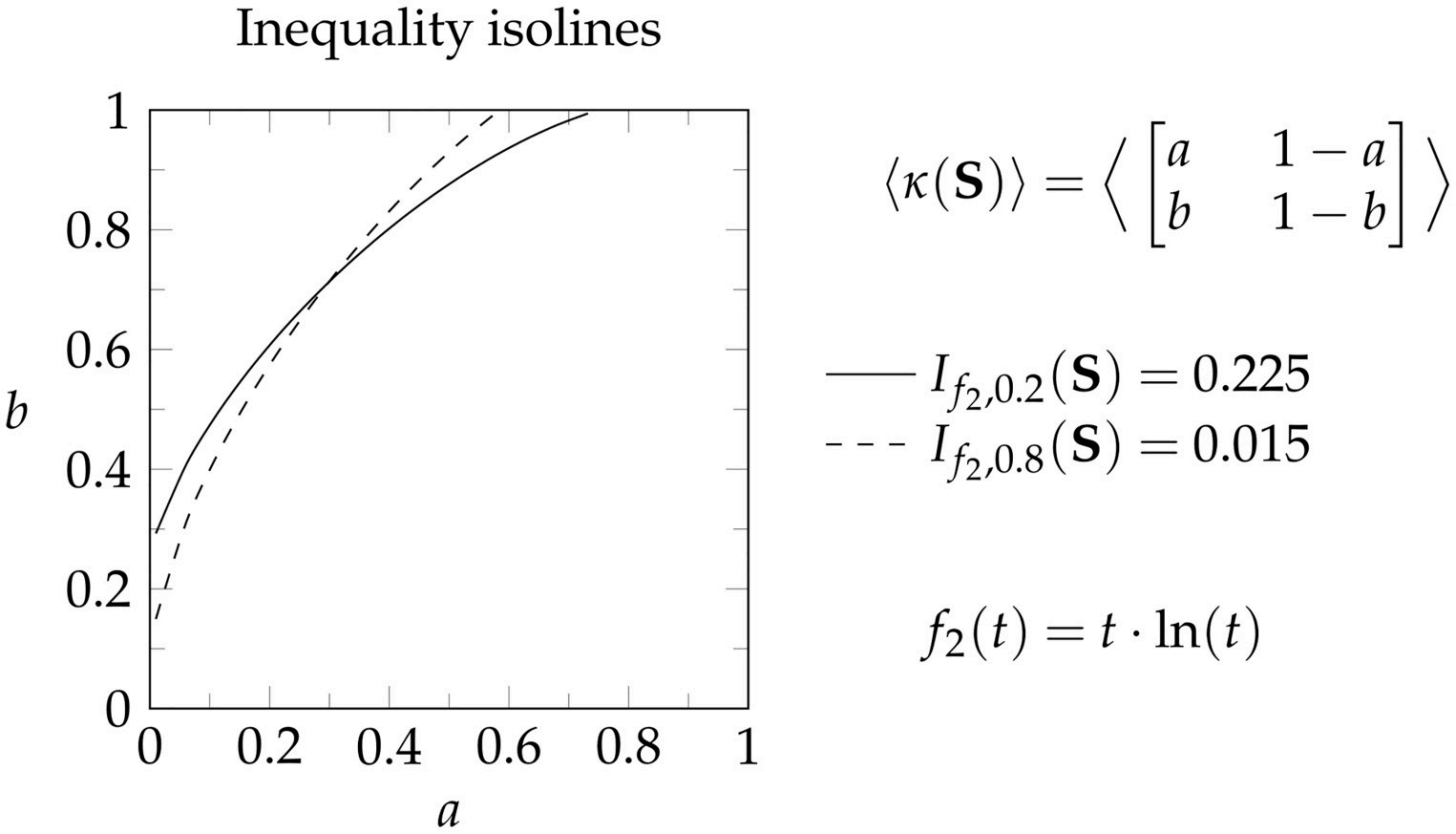
Changing the parameter *p* leads a different inequality measure. Using the same generator function *f*_2_ and different parameters *p*, we generate two isolines that indicate which 2 × 2 population matrices obtain the same inequality index. From both isolines intersecting, we can see that Lorenz incomparable populations are ranked differently depending on the chosen parameter *p*. Therefore, each parameter *p* provides a distinct inequality measure.

**Example 6**. *Consider the set of populations that can be represented by a 2 × 2 normalized population matrix. As shown in* Fig 5, *this matrix κ*(**S**) *has two parameters* (*a*, *b*). *We arbitrarily choose the generator f*_2_ = *t* ln(*t*) *and the values p* = 0.2 *and p* = 0.8. *We refer to an isoline as plot that visualized which parameter combinations* (*a*, *b*) *result in the same inequality score. Since the isolines of both inequality measures intersect* (*see* Fig 5) *the measures rank Lorenz incomparable populations differently. Thus, changing the parameter p results in a new inequality measure that is not equivalent to the original and provides an alternative way of ranking populations consistently with the required properties*.

### Preliminary intuition for redundancy and synergy

#### Intuition examples

**Fig 6:**
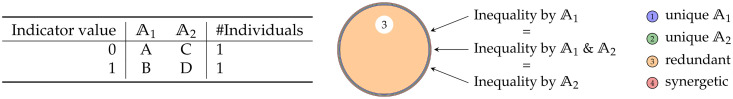
Redundancy example. Fully redundant contribution by both attributes.

Before constructing the desired decomposition, this section shall give an intuition for the concepts of redundant, unique, and synergetic contributions in the context of inequality measures. For this, we adopt typical examples [31] that are fully determined by Property M1-M5:

**Example 7** (Redundant contributions). Fig 6 *provides a fully redundant model* **M**. *Attribute* $A_{2}$ *is a re-labeled copy of attribute* $A_{1}$, *and re-labeling groups shall not affect inequality (Property M1). Therefore, the partitionings* Γ({1}, **M**), Γ({2}, **M**), *and* Γ({1, 2}, **M**) *must obtain the same inequality index as visualized by the Venn diagram in* Fig 6. *Since all regions fully intersect, we say the inequality is contributed redundantly by attributes* $A_{1}$ *and* $A_{2}$.

**Fig 7:**
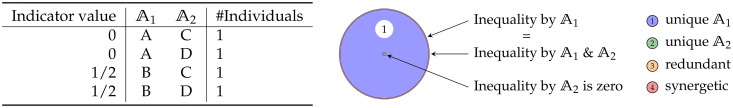
Unique example. Fully unique contribution by attribute $A_{1}$.

**Example 8** (Unique contributions). Fig 7 *provides a fully unique model* **M** *to attribute* $A_{1}$. *The partitioning* Γ({2}, **M**) *provides a uniform distribution and, thus, an inequality index of zero (Property M5). The partitioning on attribute* $A_{1}$ *provides the same population as partitioning on both attributes:* Γ({1, 2}, **M**) = Γ({1}, **M**). *Therefore, both partitionings must obtain the same inequality index. This results in the Venn diagram in* Fig 6 *and we conclude that inequality is contributed uniquely by attribute* $A_{1}$.

**Fig 8:**
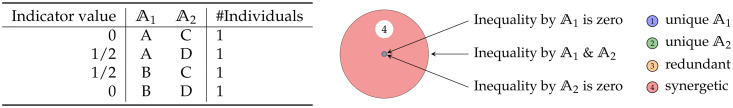
Synergetic example. Fully synergetic contribution by both attributes.

**Example 9** (Synergetic contributions). Fig 8 *provides a fully synergetic model* **M**. *Partitioning on either attribute individually* (Γ({1}, **M**) *and* Γ({2}, **M**)) *provides a uniform distribution and thus an inequality index of zero (Property M5). Non-zero inequality can only be measured when partitioning on both attributes* (Γ({1, 2}, **M**)), *which results in the Venn diagram in* Fig 8. *Therefore, we say the inequality is contributed synergetically by both attributes*.

#### Game theoretic synergy is insufficient

As it could already be seen (Eq (2) in Section “Introduction” and the previous examples), the desired attribute decomposition builds on Assumption 2:

**Assumption 2.** *Inequality can be decomposed into non-negative redundant, unique, and synergetic contributions as indicated by* Eq (34) *and* Fig 1B *for the case of two attributes*.

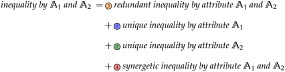
(34a)

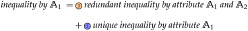
(34b)

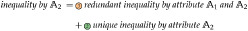
(34c)

The corresponding decomposition for Assumption 2 is challenging since it requires quantifying four partial contributions, while only three cumulative contributions can be measured (Γ({1}, **M**), Γ({2}, **M**), and Γ({1, 2}, **M**)). The resulting system of equations is under-determined, which causes the necessity of extending the inequality measure to either a notion of intersection or union. The examples in Section “Intuition examples” avoided this issue by only discussing special cases where Property M1-M5 imply that the redundant or synergetic contribution must be zero.

A (different) notion of synergy is already well established in game theory and the computation of Shapley values. Since Shapley values can be applied to inequality measures [32], it raises the question of how the challenges mentioned above have been addressed in this setting:

**Definition 13** (Game synergy and Shapley values [33, 34]). *Game synergy is a function* GS:P({1,..,n})→R, *that takes a set of attribute indices and quantifies their synergy as shown in* Eq (35) *in its direct application to this setting*.

$$\begin{array}{c} \text{GS}(a)≔\sum_{\emptyset \neq b\subseteq a}{\left(-1\right)}^{\left|a|-|b\right|}I_{f,p}\left(\Gamma \left(b,M\right)\right) \end{array}$$ (35)

*Game synergy can be used to compute Shapley values* φ:{1,..,n}→R (Eq (37)), *which shall quantify the contribution of attribute i*.

$$\begin{array}{c} \varphi (i)≔\sum_{a\in P(\{1,..,n\}\backslash \{i\})}\frac{\text{GS}(a\cup \{i\})}{|A|+1} \end{array}$$ (36)

Game synergy does not consider the concept of redundancy and thus fails to separate it from synergy, as shown in Eq (37). From our perspective, ‘game synergy’ is the difference between synergy and redundancy.

$$\begin{array}{cc} & \;\;\;\;\text{GS}\left(\left\{1,2\right\}\right)=I_{f,p}\left(\Gamma \left(\left\{1,2\right\},M\right)\right)-I_{f,p}\left(\Gamma \left(\left\{1\right\},M\right)\right)-I_{f,p}\left(\Gamma \left(\left\{2\right\},M\right)\right) \end{array}$$ (37a)

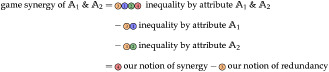
(37b)

The interpretation of Eq (37) can be used to explain the negativity of game synergy and its consequent meaning: we can interpret positive ‘game synergy’ as indication of dominant synergetic interactions between attributes, while negative ‘game synergy’ indicates dominant redundant interactions. Since both components may be present simultaneously (visualized in Fig 1B) and in a canceling direction (highlighted in Eq (37)), it would be desirable to separate them. This would enable more detailed analyses and a more practical operational interpretation, as shown in Section “Decomposing f-inequality”.

**Remark 4**. *An equivalent argument was made by Williams and Beer* [3] *for interaction information and motivated the research area of PartialInformationDecompositions*.

With this interpretation of game synergy, we can also provide an interpretation of Shapley values, as shown in Eq (38). At two attributes, the shapely value of each attribute corresponds to its unique contribution plus half of their redundancy and synergy. As a result, the Shapley values of each attribute sum to the total amount: *I*_*f*,*p*_(Γ({1, 2}, **M**)) = *φ*(1) + *φ*(2).

$$\begin{array}{cc} & \;\;\;\;\varphi \left(1\right)=\frac{\text{GS}(\{1\})}{1}+\frac{\text{GS}(\{1,2\})}{2} \end{array}$$ (38a)

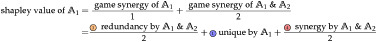
(38b)

This section provided an intuition for the desired concepts of redundancy and synergy. We highlighted the necessity of extending inequality measures to a notion of union or intersection since the decomposition is otherwise under-determined. We also explained our interpretation of game synergy and Shapley values and why we consider them insufficient for studying the interactions between attributes in this setting. Finally, both game synergy and Shapley values can be computed by combining the partial contributions of the following attribute decomposition, as indicated by Eq (37) and Eq (38).

### Decomposition lattice and required properties

This section presents the considered framework for an attribute decomposition and follows the general methodology of Williams and Beer [3] from Partial Information Decompositions: we consider a lattice that captures the desired subset relation for a set-theoretic intuition and discuss the required properties for a cumulative measure on this lattice. The partial contributions are then obtained from the Möbius inverse, which enforces an inclusion-exclusion relation between them.

**Definition 14** (Sources, atoms and union lattice [3, 35]).

- *An attribute set* a∈P({1,..,n}) *is a subset of all attribute indices that is used to construct a partition*.
  *For example:* Γ(**a**, **M**).
- *An atom* α∈A(n) *is a non-empty set of attribute sets defined by* Eq (39a). *The cardinality of* A(n) *is one less than the n-th Dedekind number* [36]. *In this work, we use atoms to represent a notion of union. For example, the atom α* = {{1}, {2, 3}} *shall represent the union of inequality when partitioning on attribute* $A_{1}$ *and* $\left(A_{2},A_{3}\right)$.
  $$\begin{array}{c} A(n)≔\left\{\alpha \in P_{1}\left(P\left(\left\{1,..,n\right\}\right)\right)\;:\;\left(\forall a,b\in \alpha \right)\left[\neg \left(a\subset b\right)\right]\right\} \end{array}$$ (39a)
  $$\begin{array}{c} (\alpha ⪯\beta )≔\left(\forall a\in \alpha .\;\exists b\in \beta .\;a\subseteq b\right) \end{array}$$ (39b)
  $$\begin{array}{c} (\alpha ⪯\beta )≔\left(\alpha ⪯\beta \;\;and\;\;\neg (\beta ⪯\alpha )\right) \end{array}$$ (39c)
- *The set of atoms form a distributive lattice with the ordering of* Eq (39b). *We refer to the resulting lattice* (A(n),⪯) *as union lattice* [36–38].

**Remark 5**. *We treat the union lattice as reversed synergy lattice. This enables the direct application of our results from* [4].

**Notation 9**.

- *We notate the meet and join on the union lattice as α* ⋏ *β and α* ⋎ *β respectively*.
- *We notate the bottom and the top of the union lattice as* ⊥_∪_ = {∅} *and* ⊤_∪_ = {{1, .., *n*}} *respectively*.
- *We notate the upset and strict upset of on the union lattice as* ↑*α and* $\dot{↑}\alpha$ *respectively*.

**Fig 9:**
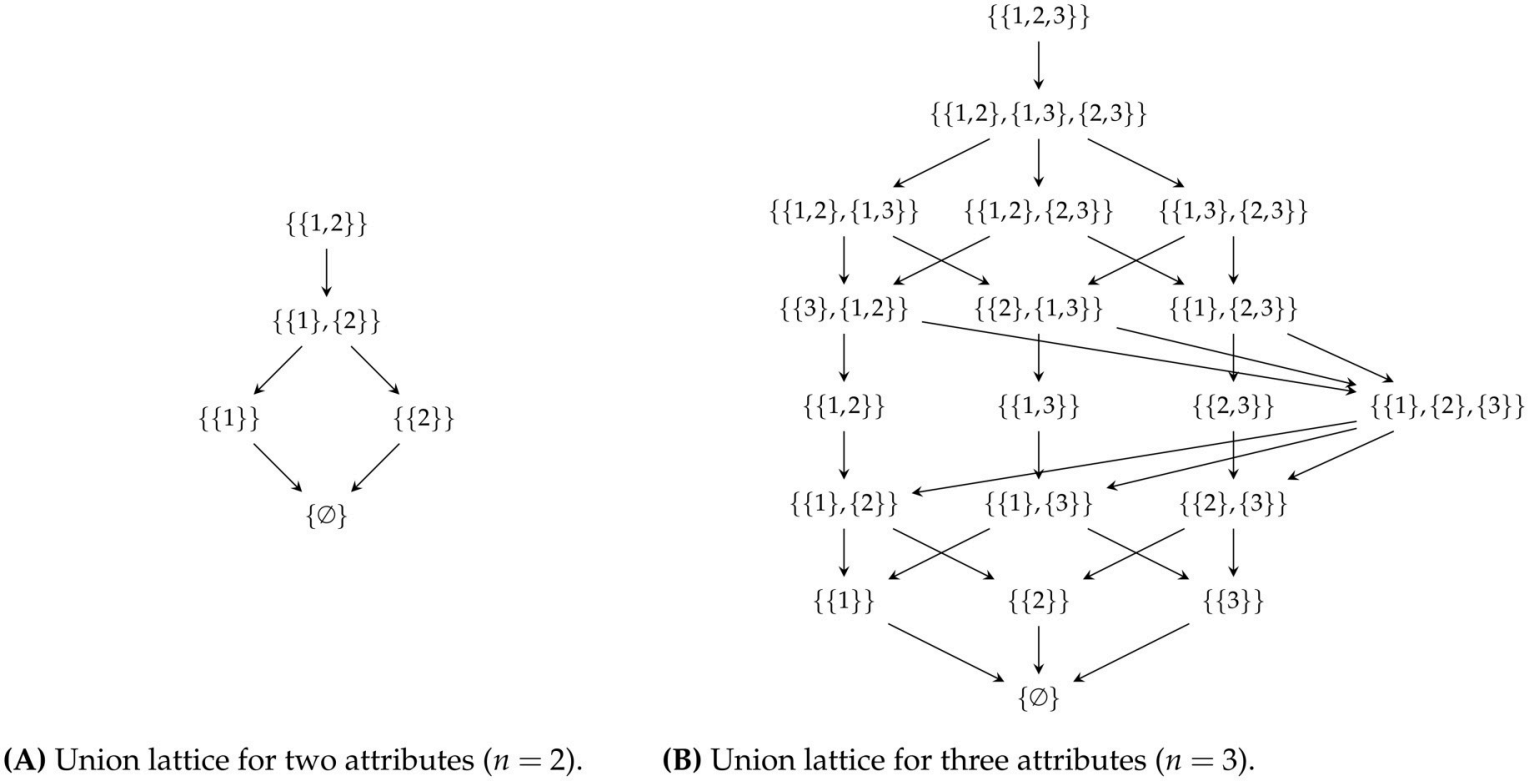
Decomposition lattice visualization. The union lattice for (A) two attributes and (B) three attributes.

The union lattice for two and three attributes is visualized in Fig 9.

Using an inequality measure *I*(⋅), we can already quantify the inequality for an attribute set **a** ∈ *α* as *I*(Γ(**a**, **M**)). However, this provides fewer equations than free variables when calculating partial contributions (under-determined) as discussed in Section “Game theoretic synergy is insufficient”. To fully determine the system, we have to extend inequality measures from attribute sets to atoms (cumulative measure) and can express partial contributions as computation on the decomposition lattice (partial measure) [3]. We first introduce both definitions and then discuss the required properties for achieving the desired set-theoretic analogy:

**Definition 15** (Cumulative measure: union inequality *I*^∪^). *The union inequality I*^∪^(*α*, **M**) *is a function that assigns a real value to every atom of the union lattice. It is a cumulative measure that shall satisfy Property U1-U4 defined below*.

**Definition 16** (Partial inequality contributions *I*^*δ*^). *The partial inequality (redundant, unique, synergetic) contributions I*^*δ*^(*α*, **M**) *are defined by the Möbius inverse* [3, 39] *on the reversed lattice* [4] *as shown in* Eq (40).

$$\begin{array}{c} I^{\cup }\left({⊤}_{\cup },M\right)-I^{\cup }\left(\alpha ,M\right)≔\sum_{\beta \in ↑\alpha }I^{\delta }\left(\beta ,M\right) \end{array}$$ (40a)

$$\begin{array}{c} I^{\delta }\left(\alpha ,M\right)=I^{\cup }\left({⊤}_{\cup },M\right)-I^{\cup }\left(\alpha ,M\right)-\sum_{\beta \in \dot{↑}\alpha }I^{\delta }\left(\beta ,M\right) \end{array}$$ (40b)

The following properties for a cumulative measure are typically presented as axioms in the context of Partial Information Decompositions [3, 40] and can directly be transferred to inequality measures:

**Property U1** (Commutativity [3, 40]). *A notion of union inequality is invariant to the order of attribute sets. Let σ*: *α* → *α permute the order of attribute sets in an atom*.

$$\begin{array}{cc} \forall \alpha \in A(n): & I^{\cup }\left(\alpha ,M\right)=I^{\cup }\left(\left\{\sigma \left(a\right)\;:\;a\in \alpha \right\},M\right) \end{array}$$ (41a)

$$\begin{array}{cc} \text{Example}: & I^{\cup }\left(\left\{\left\{1\right\},\left\{2,3\right\}\right\},M\right)=I^{\cup }\left(\left\{\left\{2,3\right\},\left\{1\right\}\right\},M\right) \end{array}$$ (41b)

**Property U2** (Monotonicity [3, 40]). *Adding an attribute set to an atom can only increase their union inequality:*

$$\begin{array}{cc} \forall \alpha \in A(n),\;\forall a\in P(\{1,..,n\}): & I^{\cup }\left(\alpha ,M\right)\leq I^{\cup }\left(\alpha \cup \left\{a\right\},M\right) \end{array}$$ (42a)

$$\begin{array}{cc} \text{Example}: & I^{\cup }\left(\left\{\left\{2,3\right\}\right\},M\right)\leq I^{\cup }\left(\left\{\left\{2,3\right\},\left\{1\right\}\right\},M\right) \end{array}$$ (42b)

**Property U3** (Self-inequality [3, 40]). *The union of a single attribute set equals the desired inequality measure*.

$$\begin{array}{cc} \forall a\in P(\{1,..,n\}): & I^{\cup }\left(\left\{a\right\},M\right)=I\left(\Gamma \left(a,M\right)\right) \end{array}$$ (43a)

$$\begin{array}{cc} \text{Example}: & I^{\cup }\left(\left\{\left\{2,3\right\}\right\},M\right)=I\left(\Gamma \left(\left\{2,3\right\},M\right)\right) \end{array}$$ (43b)

**Property U4** (Non-negativity [3, 40]). *The partial inequality contributions are non-negative*.

$$\begin{array}{cc} \forall \alpha \in A(n): & I^{\delta }\left(\alpha ,M\right)\geq 0 \end{array}$$ (44)

**Fig 10:**
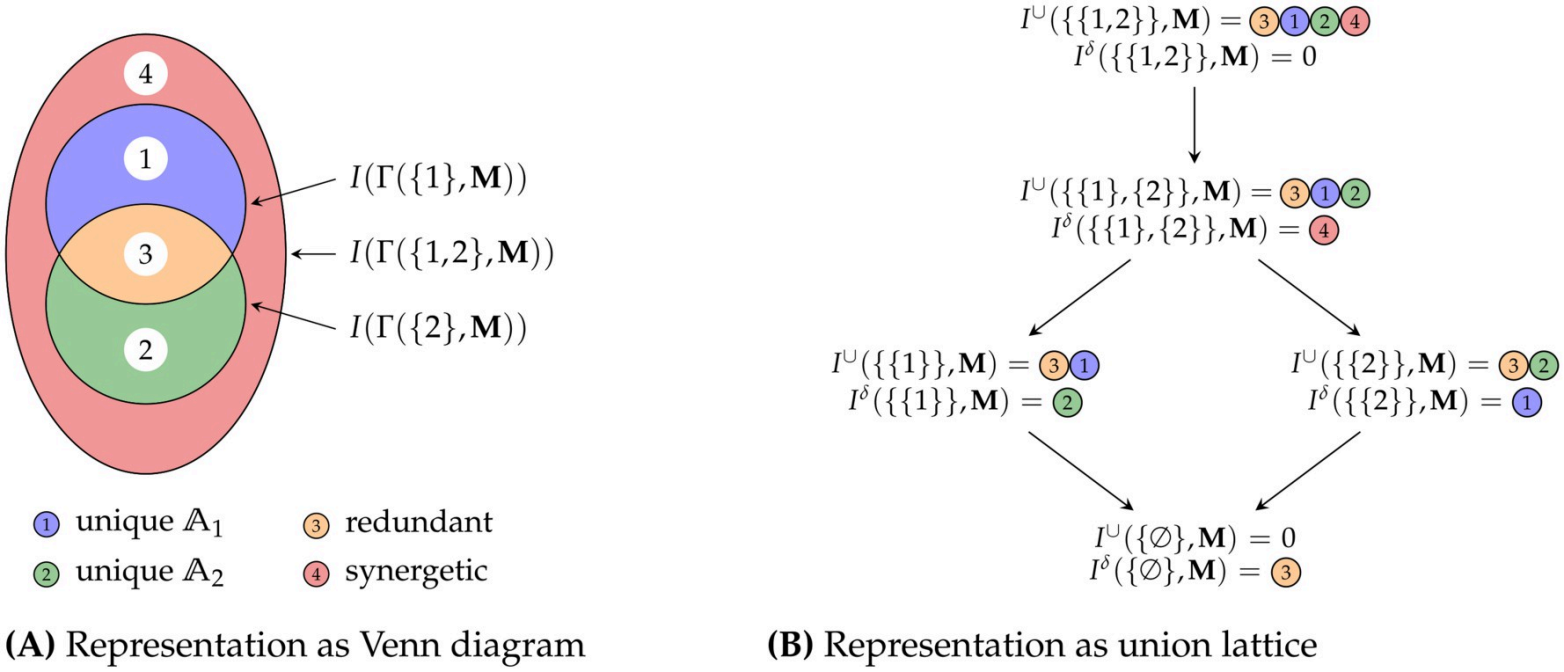
Visualization for the relation between Venn diagrams and the union lattice at two attributes (*n* = 2). Representation of partial contributions as (A) Venn diagram and (B) union lattice. The partial contribution of the top element is always zero. All other partial contributions of an atom on the union lattice (*I*^*δ*^) correspond to exactly one partial region in the Venn diagram.

The combination of Property U2 and the union lattice ensures the expected subset relation. Property U3 binds the union measure to the desired inequality measure. Property U4 ensures the interpretability of results by enabling the analogy from a population’s inequality to a set’s cardinality. Finally, Fig 10 visualizes the relation between a Venn diagram and the used decomposition lattice at the example of *n* = 2. Except for the top element, each partial contribution on the union lattice *I*^*δ*^(⋅, **M**) corresponds to a partial region of the Venn diagram.

This section discussed the considered decomposition framework based on the union lattice and the necessary properties for the required cumulative measure.

**Remark 6**. *If desired, the decomposition on the union lattice can be transformed into a decomposition on the redundancy lattice* [3] *as described in* [4, *Section 3.4*].

**Remark 7**. *The attribute decomposition presented in this work is applicable to any finite number of attributes. However, the number of interactions between attributes (colored regions in the Venn diagram of* Fig 1) *grows super-exponentially with the number of attributes. This causes a limit in practice*.

*We provide examples using two attributes throughout this work to illustrate the underlying concepts. At more than four attributes, we recommend iteratively exploring specific interactions of interest rather than attempting to compute the full decomposition*.

### Decomposing f-inequality

With the decomposition framework of Section “Decomposition lattice and required properties”, we only have to define a union inequality measure ($I_{f,p}^{\cup }$) for *f*-inequality to obtain its decomposition. To achieve the required properties and a practical operational interpretation, we use the join of the zonogon order (convex-hull), as shown in Definition 17. Intuitively, this appears suitable since it reflects the unique and measure independent optimal dependence between attributes as a notion of their union.

**Definition 17** (*f*-inequality union). *We define the union of two partitions by their join under the zonogon order*.

$$\begin{array}{c} I_{f,p}^{\cup }\left(\alpha ,M\right)≔I_{f,p}(\underset{a\in \alpha }{⊔}\left\langle \Gamma \left(a,M\right)\right\rangle ) \end{array}$$ (45)

**Theorem 3**. *Definition 17 satisfies Property U1-U4*.

The proof of Theorem 3 is shown in S3 Appendix.

**Remark 8**. S4 Appendix *shows that we can compute partial contributions in a practical implementation using* Eq (46), *where* C(·) *is the n-ary Cartesian product. We recommend caching the cumulative measure I*^∪^(⋅, **M**) *to avoid repeated computations. This implementation is advantageous by computing the Möbius inverse without having to identify and visit each element in the strict upset of an atom* ($\dot{↑}\alpha$) *as the lattice* |A(n)| *grows rapidly in n*.

$$\begin{array}{c} \text{reduce}(∼,\alpha )=\{a\in \alpha \;:\;\neg (\exists b\in \alpha )[a∼b]\} \end{array}$$ (46a)

$$\begin{array}{c} \text{dual}(\alpha )=\text{reduce}(⊃,\;C(\{\{1,..,n\}\backslash a\;:\;a\in \alpha \})) \end{array}$$ (46b)

$$\begin{array}{cc} I_{f,p}^{\delta }\left(\alpha ,M\right) & =\{\begin{array}{cc} 0 & if\;\alpha ={⊤}_{\cup }\\ \sum_{\beta \in P(\text{dual}(\alpha ))}{\left(-1\right)}^{|\beta |-1}\;I_{f,p}^{\cup }\left(\text{reduce}\left(\subset ,\;\alpha \cup \beta \right),M\right) & otherwise \end{array} \end{array}$$ (46c)

The resulting operational interpretation depends on the type of *f*-inequality:

$$\begin{array}{c} \begin{array}{cc} \text{synergetic}\;\text{contribution}\;\; & \begin{array}{c} \overset{\text{weak}}{\Rightarrow }\\ \underset{\text{strong}}{\Leftrightarrow } \end{array}\;\;\text{sub-optimal}\;\text{dependence}\;\text{between}\;\text{attributes}\\ \text{unique}\;\text{contribution}\;\; & \begin{array}{c} \overset{\text{weak}}{\Rightarrow }\\ \underset{\text{strong}}{\Leftrightarrow } \end{array}\;\;\text{no}\;\text{Pigou-Dalton}\;\text{transfers}\;\text{from}\;\text{other}\;\text{attribute} \end{array} \end{array}$$

Synergetic contributions indicate that inequality can be reduced by re-distributing the indicator variable based on the dependence between attributes or suitably increasing the dependence between attributes. Unique contributions can be reduced by re-distributing the indicator variable based on the specific attribute or changing the distribution of this attribute. As it can be seen from Corollary 2, the resulting notion of redundancy is lower bound by the quantification of the zonogon meet (intersection).

**Fig 11:**
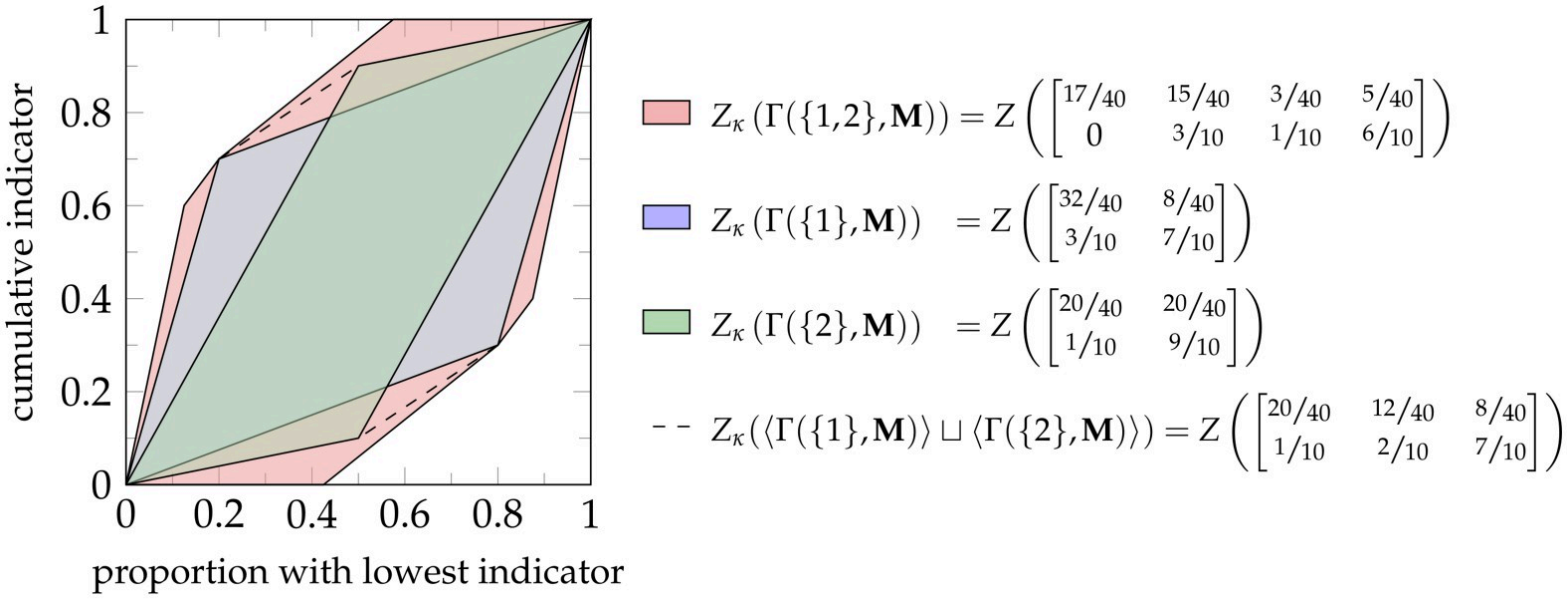
Visualization of the model from Table 2.

**Example 10**. *Consider the model* **M** *obtained from* Table 2 *with the two attributes* $A_{1}=\left\{A,B\right\}$ *and* $A_{2}=\left\{C,D\right\}$. *The corresponding population matrices and zonogons for model* **M** *are visualized in* Fig 11.

**Table 2:** Example population model.

Indicator value	$A_{1}$	$A_{2}$	Number of individuals
0	A	C	170
3150	A	D	150
130	B	C	30
650	B	D	50

*To analyze the model, we first define an inequality measure that suitably captures the required properties for the specific application. This is important since it determines how (zonogon) incomparable populations shall be ranked. For f-inequality, this is determined by the* (*f*, *p*) *combination. Assume we consider the population* Γ({2}, **M**) *preferable over* Γ({1}, **M**) *and thus want to assign it a smaller inequality index. Without further information, we arbitrarily choose the inequality measure obtained from Definition 12 using the χ*^2^-*divergence f*(*t*) = (*t*−1)^2^ *with p* = 0.4, *as shown in* Eq (47).

Iχ2,0.4S=∑v→∈κSrv→where:r([]yx)=0.9·x−y2x+1.5·y=1S¯S∑s∈S0.9·S¯−s2S¯+1.5·s (47)

**Fig 12:**
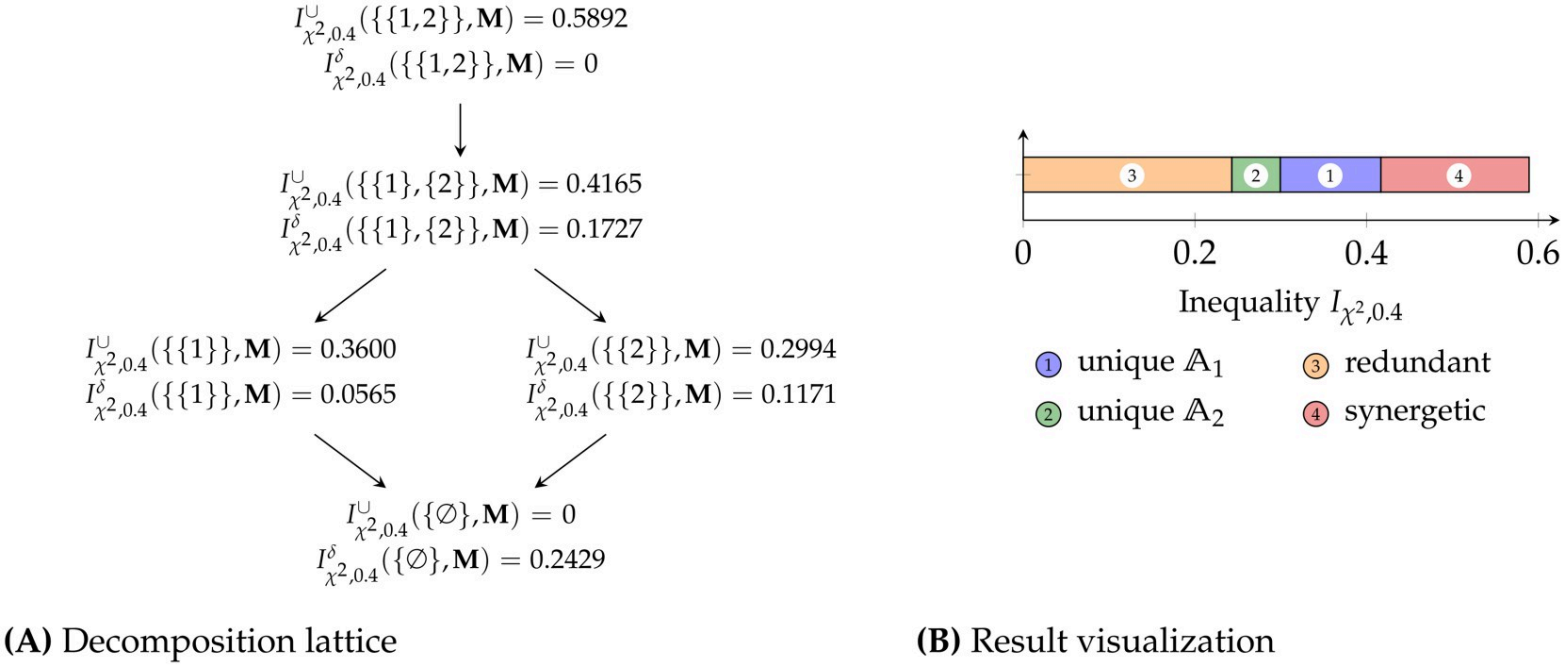
Result visualization (rounded). Considering the model from Table 2 with the inequality measure of Eq (47).

*We can compute the attribute decomposition using Definition 17 and* Eq (40) *or* Eq (46). *The results are visualized in* Fig 12.

### Decomposing the Atkinson index

The presented attribute decomposition can be extended to any invertible transformation of an *f*-inequality. We demonstrate the approach using the Atkinson index (Eq (12) with *d* ∈ (0, 1]), which is a transformation of an *f*-inequality (Generalized Entropy index, Theorem 2 and Eq (12)) as shown in Eq (48).

$$\begin{array}{cc} v_{d}\left(m\right) & =\{\begin{array}{cc} 1-e^{-m} & \text{if}\;d=1\\ 1-{\left[d\left(d-1\right)m+1\right]}^{\frac{1}{1-d}} & \text{if}\;0<d<1 \end{array} \end{array}$$ (48a)

$$\begin{array}{c} A_{d}\left(S\right)=v_{d}\left({\text{GE}}_{1-d}\left(S\right)\right) \end{array}$$ (48b)

The transformation function *v*_*d*_ correctly maintains the bottom element at zero (*v*_*d*_(0) = 0) and is invertible: the case of *d* = 1 is invertible, and the case of 0 < *d* < 1 is invertible for the required domain shown in Eq (49). Therefore, we can view the Atkinson index as a re-graduation [41] on the decomposition lattice.

$$\begin{array}{cc} 0 & <d<1 \end{array}$$ (49a)

$$\begin{array}{c} {\text{GE}}_{1-d}\left({⊥}_{S}\right)={\text{GE}}_{1-d}\left(\left\{1\right\}\right)=0\leq m\leq \frac{1-2^{-d}}{d-d^{2}}={\text{GE}}_{1-d}\left(\left\{1,0\right\}\right)={\text{GE}}_{1-d}\left({⊤}_{S}\right) \end{array}$$ (49b)

The attribute decomposition of the Atkinson index is obtained by Eq (50) [4, Section 3.6], which maintains the operational interpretation of the decomposition from the Generalized Entropy index.

$$\begin{array}{cc} f_{c}\left(t\right)≔ & \left\{ \begin{array}{cc} t\;\text{ln}(t) & \text{if}\;c=0\\ \frac{t^{1-c}-t}{(c-1)c}\; & \text{if}\;0<c<1 \end{array}\right. \end{array}$$ (50a)

$$\begin{array}{c} A_{d}^{\cup }\left(\alpha ,M\right)≔v_{d}\left(I_{f_{1-d},0}^{\cup }\left(\alpha ,M\right)\right) \end{array}$$ (50b)

$$\begin{array}{c} A_{d}^{\delta }\left(\alpha ,M\right)≔v_{d}\left(I_{f_{1-d},0}^{\delta }\left(\alpha ,M\right)\right) \end{array}$$ (50c)

The resulting re-graduation of the decomposition lattice satisfies the inclusion-exclusion relation (Möbius inverse, Eq (40)) under a transformed definition of addition shown in Definition 18. This corresponds to the addition under which the partial contributions sum to the total amount. Moreover, the transformation maintains the desired Properties U1-U4 [4, Section 3.6].

**Definition 18** (Addition of Atkinson index inequality). *We define the addition* ⊕_*d*_ *and subtraction* ⊖_*d*_ *on the cumulative and partial Atkinson index* ($A_{d}^{⋄}\left(S\right)$ *where* ⋄ ∈ {*δ*, ∪}) *by:*

$$\begin{array}{c} A_{d}^{⋄}\left(S_{1}\right){⊕}_{d}A_{d}^{⋄}\left(S_{2}\right)≔\;\;v_{d}\left(v_{d}^{-1}\left(A_{d}^{⋄}\left(S_{1}\right)\right)+v_{d}^{-1}\left(A_{d}^{⋄}\left(S_{2}\right)\right)\right) \end{array}$$ (51a)

$$\begin{array}{c} A_{d}^{⋄}\left(S_{1}\right){⊖}_{d}A_{d}^{⋄}\left(S_{2}\right)≔\;\;v_{d}\left(v_{d}^{-1}\left(A_{d}^{⋄}\left(S_{1}\right)\right)-v_{d}^{-1}\left(A_{d}^{⋄}\left(S_{2}\right)\right)\right) \end{array}$$ (51b)

An interesting observation is that combining the resulting partial contributions into a Shapley value (Eq (38) using the addition of Definition 18) is equivalent to computing the Shapley value directly from Definition 13 and Definition 18. This indicates that the addition used when computing Shapley values should depend on the used inequality measure to maintain consistency between their results.

### Multi-layered inequality

In practical scenarios, inequality may appear in different layers, such as income, healthcare, or housing. As discussed by Andreoli and Zoli [20], this setting provides two options for aggregating multidimensional distributions: a) first aggregating the layers to represent each individual by a single value and then applying an inequality measure to the result, or b) first aggregating each layer using an inequality measure and then combining the inequality results of each layer. The latter approach has the disadvantage that the dependence between layers (e.g. dependence between income and housing) has no affect the final result [20, p 20].

As we discuss next, any multi-layered inequality measure can directly be attribute decomposed if the aggregation is a linear combination of *f*-inequalities or their transformation, as long as, the used addition and multiplication is consistent with the measure. This allows for using different *f*-inequalities on each layer to precisely specify which distributions are more desirable and restricts Pigou-Dalton transfers within each layer. While this is easily decomposable, the resulting measure is not affected by the dependence between indicators.

**Definition 19**. *A multi-layered inequality model is a set of models* $M=\{M_{i}:1\leq i\leq k\}$ *which share the same attributes and individuals. This provides k indicator values for each individual*.

**Definition 20** (Layered *f*-inequality). *Let* $S=\left\{S_{i}\;:\;1\leq i\leq k\right\}=\left\{\Gamma \left(a,M_{i}\right)\;:\;M_{i}\in M\right\}$ *be a set of k populations obtained from a set of attributes* **a** *and a multi-layered inequality model* M. *We define a layered inequality measure* I(S) *as weighted sum* (*w*_*i*_ ≥ 0) *of f-inequality measures. Like indicated by the subscripts of f and p in* Eq (52), *the considered f-inequality can vary between layers to emphasize important characteristics of inequality on the respective layer*.

$$\begin{array}{c} I(\left\{S_{1},\ldots ,S_{k}\right\})≔\sum_{i=1}^{k}w_{i}I_{f_{i},p_{i}}\left(S_{i}\right) \end{array}$$ (52)

**Definition 21** (Layered *f*-inequality decomposition). *Since the partial contributions of any f-inequality consider the same notion of addition, their decompositions are additive as shown in* Eq (53). *The resulting decomposition restricts Pigou-Dalton transfer to each layer and satisfies an inclusion-exclusion relation (Möbius inverse)*.

$$\begin{array}{cc} I^{\cup }\left(\alpha ,M\right)≔ & \sum_{i=1}^{k}w_{i}I_{f_{i},p_{i}}^{\cup }\left(\alpha ,M_{i}\right) \end{array}$$ (53a)

$$\begin{array}{cc} I^{\delta }\left(\alpha ,M\right)≔ & \sum_{i=1}^{k}w_{i}I_{f_{i},p_{i}}^{\delta }\left(\alpha ,M_{i}\right) \end{array}$$ (53b)

**Remark 9**. *The ideal attribute dependence can vary between indicator values. Therefore, there may not exist an attribute dependence that leads to zero synergy for the layered measure*.

The notion of addition for the partial contributions has to be identical on each layer to obtain an inclusion-exclusion relation on the layered measure. To maintain this consistency when transforming layered inequality, the same transformation has to be applied to all layers.

**Notation 10** (Transformed addition and multiplication). *Consider a specific invertable transformation v*(⋅), *then we obtain the following notion of addition and multiplication on transformed inequality measures:*

$$\begin{array}{c} a⊕b≔\;\;v(v^{-1}\left(a\right)+v^{-1}\left(b\right)) \end{array}$$ (54a)

$$\begin{array}{c} a⊙b≔\;\;v(v^{-1}\left(a\right)\;\cdot \;v^{-1}\left(b\right)) \end{array}$$ (54b)

Transforming a layered inequality measure equals transforming each *f*-inequality and their corresponding operators as shown in Eq (55) by Definition 21 and Notation 10. As a result, the inclusion-exclusion relation (Möbius inverse) of partial contributions from the transformed measure is maintained under the transformed addition operation.

$$\begin{array}{c} v(I^{\cup }\left(\alpha ,M\right))=v\left(w_{1}\right)⊙v\left(I_{f_{1},p_{1}}^{\cup }\left(\alpha ,M_{1}\right)\right)\;⊕\;\ldots \;⊕\;v\left(w_{k}\right)⊙v\left(I_{f_{k},p_{k}}^{\cup }\left(\alpha ,M_{k}\right)\right) \end{array}$$ (55a)

$$\begin{array}{c} v(I^{\delta }\left(\alpha ,M\right))=v\left(w_{1}\right)⊙v\left(I_{f_{1},p_{1}}^{\delta }\left(\alpha ,M_{1}\right)\right)\;⊕\;\ldots \;⊕\;v\left(w_{k}\right)⊙v\left(I_{f_{k},p_{k}}^{\delta }\left(\alpha ,M_{k}\right)\right) \end{array}$$ (55b)

### The relation of inequality and information

This section brings the results from decomposing inequality into the context of decomposing information. Their relation is intuitive since both information and inequality measures aim to quantify a notion of distance from the uniform distribution. We think studying their relation provides valuable insights and can encourage the transfer of results between areas.

A Partial Information Decomposition [3, 42] aims to understand how a set of source variables provides information redundantly, uniquely or synergistically about a target. We use the following notation:

**Notation 11**.

- *We notate a discrete visible/source variable V with state v in the state space* $V=\{v_{1},...,v_{m}\}$ .
- *We notate a discrete target variable T with state t in the state space* T .
- *We notate an indicator variable for state t of variable T as* **1**_*T*_(*t*).

We can define discrete *f*-information as shown in Eq (56) [4, Section 3.1]. Notice that Eq (56b) is *f*-inequality as defined in Section “Defining f-inequality” and that *f*-information is its expected value (Eq (56c)). Therefore, discrete *f*-information is a layered inequality measure by Definition 20. We refer to *i*_*f*,*p*_(*κ*) as specific or target-pointwise information. The ordering of populations by the Atkinson criterion is identical to the ordering of random variables by the Blackwell order with respect to an indicator variable [29, 43], which is the binary target **1**_*T*_(*t*). In this context, the zonogon *Z*_*κ*_(*P*(*V*∣**1**_*T*_(*t*))) represents the achievable trade-off between the type-I and type-II error for predicting the state t∈T [4] and its lower boundary is known as Neyman-Pearson boundary [28, Section 14.1]. The transformation of measures is also used in both areas: just like the Atkinson index is an invertible transformation on an *f*-inequality, so is Rényi-information an invertible transformation of an *f*-information [4]. Due to these relations, the presented methodology in this work can directly be applied to obtain non-negative Partial Information Decompositions with practical operational interpretation, as shown in [4].

$$P\left(V|1_{T}\left(t\right)\right)≔\left[\begin{array}{lll} p\left(V=v_{1}|T=t\right) & \cdots & p\left(V=v_{m}|T=t\right)\\ p(V=v_{1}|T\neq t) & \cdots & p(V=v_{m}|T\neq t) \end{array}\right]$$ (56a)

$$\begin{array}{cc} i_{f,p}(\kappa )≔ & \sum_{\vec{v}\in \kappa }r_{f,p}\left(\vec{v}\right) \end{array}$$ (56b)

$$\begin{array}{c} \text{discrete}\;f\text{-information}\;\text{of}\;(V;T)≔E_{t\in T}\left[i_{f,p(T=t)}\left(P\left(V|1_{T}\left(t\right)\right)\right)\right] \end{array}$$ (56c)

This creates a relation between some commonly used information and inequality measures, as shown in Table 3. It may be desirable to survey existing inequality measures in the future to see if they are (invertible transformations of) an *f*-inequality and identify the equivalent (transformation of an) *f*-information.

**Table 3:** Relation of commonly used inequality and information measure.

*f*-information measure	Generator	*f*-inequality measure
Total Variation	*f*(*t*) = 0.5|*t*−1|	Pietra index
Reversed Kullback-Leiber-Information	*f*(*t*) = −ln(*t*)	Theil index/Generalized Entropy (*c* = 1)
Mutual Information	*f*(*t*) = *t* ln(*t*)	Generalized Entropy (*c* = 0)

Some key relations between both areas are summarized in Table 4. Consequently, we see further opportunities to apply concepts and insights from one area to the other. In particular, we are curious about the resulting interpretation when applying subgroup decompositions from inequality measures to specific information. Moreover, understanding interactions due to dependencies between multiple layers for inequality measures may provide new insights for the information decomposition at multiple targets.

**Table 4:** Equivalences between measures of information and inequality.

Information Measures		Inequality Measures
Blackwell order with respect to an indicator variable	⇔	Atkinson criterion
Neyman-Pearson boundary	⇔	Lorenz curve
specific *f*-information	⇔	*f*-inequality

## Discussion

In this work, we extended methods used in information theory to provide a new perspective for the decomposition of inequality from economics and social science. We introduced a class of inequality measures (*f*-inequalities), which are based on satisfying a triangle inequality on the Lorenz curve. These measures are particularly interesting due to their mathematical properties and connection to information theory.

It remains an open question whether *f*-inequalities are the only measures that satisfy the presented properties for a decomposition as shown in Section “Decomposing f-inequality”. However, a complementing approach based on extending inequality measures through a notion of redundancy is possible: The Gini coefficient is not an *f*-inequality and can be extended by using the meet of the Atkinson order as notion of intersection on what is known as redundancy lattice [3]. The proof of non-negativity for partial contributions (Property U4) can be shown equivalently to S3 Appendix based on Eq (20). However, this approach provides a different operational interpretation for partial contributions. Thus, attribute decompositions can be constructed beyond *f*-inequalities and further research is needed.

In practical applications, it can be valuable to trace how partial inequality contributions evolve over time [9]. Although it has not been discussed in this work, this can be achieved using the method described in [4, Section 4.2].

Typical decompositions currently assume categorical attributes for forming clear partitions and subgroups. It appears to be an open research question of extending these ideas to attributes with a notion of similarity (distance) between states. For example, a person’s age in years is discrete but not categorical, which leads to a more fuzzy definition of subgroups. We think it would be desirable to better understand the treatment of such variables in both inequality and information decompositions.

We noted in Section “Decomposing the Atkinson index” that it would be desirable to utilize different notions of addition when computing Shapley values from inequality measures to ensure the consistency of results between related measures. This highlights the difficulty of transferring concepts between areas. However, we are optimistic that such issues can be avoided between inequality and information measures since they share an identical underlying representation and ordering relation (also see [44]).

Our study focused on the *interactions between attributes* to decompose to inequality, but we did not explore *interactions between indicator variables* (see “Multi-layered inequality”). This is an intriguing direction for future research, as it directly relates to the open question about interactions between target variables in information decompositions.

To evaluate multi-layered inequality, one could extend the underlying representations from two-dimensional zonogons to higher-dimensional zonoids [20, 25, 26]. However, this raises several challenges for attribute decompositions: zonoids at more than two dimensions do not form a lattice by their subset relation or multiplication with stochastic matrix [29]. As a result, they do not provide a unique underlying representation for a notion of union or intersection. Moreover, the non-negativity of the decomposition may not be achieved when extending the methodology to multivariate orderings at the considered measures froms Section “Multi-layered inequality” [37, 45]. This again highlights the importance of further research for understanding the structure of inequality caused by the dependencies between multiple indicator variables.

## Conclusions

In this work, we presented a new family of inequality measures and a new type of inequality decomposition. The presented decomposition focuses on the interactions between attributes of an individual to identify how inequality is obtained from the redundant, unique, and synergetic interactions between them. We demonstrated that the analysis by game synergy and Shapley values cannot separate the desired components and that the decomposition requires an extension of the inequality measure. We defined an extension for the introduced family of inequality measures, which satisfies the required properties and provides a practical operational interpretation. This generates a decomposition for established measures, such as the Generalized Entropy and Atkinson index. Finally, we discussed the relation between measures of information and inequality to encourage the transfer of results between both areas.

## Supporting information

S1 Appendix Relation of Property M1-M5 to the zonogon order. Discusses the representation of Property M1-M5.Proof of Lemma 1 (Property (week ⋆) implies the weak Property M1-M5).Proof of Lemma 2 (Property (strick ⋆) implies the strict Property M1-M5).
(PDF)

S2 AppendixProperties and special cases of f-inequality.
Proof of Theorem 1 (Properties of $r_{f,p}\left(\vec{v}\right)$ and *I*_*f*,*p*_(**S**)).Proof of Lemma 3 (Minkowski addition to *f*-inequality addition).Proof of Theorem 2 (Pietra index and Generalized Entropy index are special cases of f -inequality).(PDF)

S3 Appendix Decomposition properties. Proof of Theorem 3 (the constructed decomposition satisfies Property U1-U4).
(PDF)

S4 Appendix Implementation suggestion. Shows the correctness of the suggested implementation for the decomposition in Remark 8 (Section “Decomposing f-inequality”).
(PDF)
