# Supplementary material for: Quantifying redundancies and synergies with measures of inequality
Source: PLoS One. 2024 Nov 20;19(11):e0313281. doi: 10.1371/journal.pone.0313281 (PMC11578534; doi:10.1371/journal.pone.0313281)
Supplement: S1 Appendix — Discusses the representation of Property M1-M5.Proof of Lemma 1 (Property (week ⋆) implies the weak Property M1-M5).Proof of Lemma 2 (Property (strick ⋆) implies the strict Property M1-M5). (PDF) [file pone.0313281.s001.pdf]

S1 Appendix. Relation of Property M1-M5 to the zonogon order.

Representation of Property M1-M5:

1. **Label invariance** (Property M1): Re-labeling individuals and groups is the re-ordering of columns in the population matrix. This is a column permutation and, therefore, equivalent to the multiplication with a permutation matrix $\mathbf{P}$, as shown in Eq (57).

$$\kappa(\mathbf{S}_2) = \kappa(\mathbf{S}_1)\mathbf{P} \tag{57}$$

Lemma 4. *If population $\mathbf{S}_2$ is a relabeling of $\mathbf{S}_1$, then both populations are in the same equivalence class $\langle \mathbf{S}_1 \rangle = \langle \mathbf{S}_2 \rangle$.*

Proof. The inverse of a permutation matrix is its transpose ($\mathbf{P}^{-1} = \mathbf{P}^T$). Since permutation matrices are double stochastic, $\mathbf{P}$ and $\mathbf{P}^T$ are row stochastic. For any population re-labeling:

$$\begin{aligned} \kappa(\mathbf{S}_2) &= \kappa(\mathbf{S}_1)\mathbf{P} \\ \kappa(\mathbf{S}_1) &= \kappa(\mathbf{S}_2)\mathbf{P}^T \end{aligned} \tag{58}$$

Thus, we obtain:

$$\begin{aligned} Z_\kappa(\mathbf{S}_1) &= Z_\kappa(\mathbf{S}_2) && \text{(by Eq (16))} \\ \mathbf{S}_1 &\cong \mathbf{S}_2 && \text{(by Definition 7)} \\ \langle \mathbf{S}_1 \rangle &= \langle \mathbf{S}_2 \rangle && \text{(by Notation 4)} \end{aligned}$$

□

2. **Duplication invariance** (Property M2): Duplicating a population is equivalent to duplicating and normalizing the corresponding population matrix, as shown in Eq (59).

$$Z\left(\kappa(\mathbf{S} \uplus \mathbf{S})\right) = Z\left(0.5\begin{bmatrix}\kappa(\mathbf{S}) & \kappa(\mathbf{S})\end{bmatrix}\right) \tag{59}$$

Lemma 5. *Duplicating a population does not affect its equality class: $\langle \mathbf{S} \rangle = \langle \mathbf{S} \uplus \mathbf{S} \rangle$.*

Proof. Let $\mathbf{P}_1$ and $\mathbf{P}_2$ be permutation matrices to generate the desired ordering of columns for the relation of Eq (60). By Lemma 4, this does not affect their equivalence class.

$$\kappa(\mathbf{S} \uplus \mathbf{S})\mathbf{P}_2 = 0.5\begin{bmatrix}\kappa(\mathbf{S})\mathbf{P}_1 & \kappa(\mathbf{S})\mathbf{P}_1\end{bmatrix} \tag{60}$$

Let $\mathbf{I}$ be an $|\mathbf{S}| \times |\mathbf{S}|$ identity matrix, then we can find that both population matrices can be represented as multiplication of the other by a row stochastic matrix as shown in Eq (61). As discussed in Lemma 4, this implies $\langle \mathbf{S} \rangle = \langle \mathbf{S} \uplus \mathbf{S} \rangle$ by Eq (16), Definition 7, and Notation 4.

$$\kappa(\mathbf{S} \uplus \mathbf{S})\mathbf{P}_2 = \kappa(\mathbf{S})\mathbf{P}_1 \cdot 0.5 \cdot \begin{bmatrix} \mathbf{I} & \mathbf{I} \end{bmatrix} \tag{61a}$$

$$\kappa(\mathbf{S})\mathbf{P}_1 = \kappa(\mathbf{S} \uplus \mathbf{S})\mathbf{P}_2 \begin{bmatrix} \mathbf{I} \\ \mathbf{I} \end{bmatrix} \tag{61b}$$

□

3. **Scale invariance** (Property M3):

Lemma 6. *If population $\mathbf{S}_1 = \{k \cdot s_i \ : \ s_i \in \mathbf{S}_2\}$ is a linear scaling for the indicator value of $\mathbf{S}_2$ by $k \in \mathbb{R}_{>0}$, then both populations are in the same equivalence class: $\langle \mathbf{S}_1 \rangle = \langle \mathbf{S}_2 \rangle$.*

Proof.

$$\kappa(\mathbf{S}_1) = \frac{1}{|\mathbf{S}_1|} \begin{bmatrix} \begin{matrix} 1 \\ s/\overline{\mathbf{S}_1} \end{matrix} & : s \in \mathbf{S}_1 \end{bmatrix} \qquad \text{(by Definition 4)} \tag{62a}$$

$$= \frac{1}{|\mathbf{S}_2|} \begin{bmatrix} \begin{matrix} 1 \\ \frac{ks}{k\overline{\mathbf{S}_2}} \end{matrix} & : s \in \mathbf{S}_2 \end{bmatrix} \qquad \text{(by assumption)} \tag{62b}$$

$$= \frac{1}{|\mathbf{S}_2|} \begin{bmatrix} \begin{matrix} 1 \\ s/\overline{\mathbf{S}_2} \end{matrix} & : s \in \mathbf{S}_2 \end{bmatrix} \qquad \text{(by } k \neq 0\text{)} \tag{62c}$$

$$= \kappa(\mathbf{S}_2) \qquad \text{(by Definition 4)} \tag{62d}$$

$$\Longrightarrow \langle \mathbf{S}_1 \rangle = \langle \mathbf{S}_2 \rangle \qquad \text{(by Lemma 4)} \tag{62e}$$

□

4. **Pigou-Dalton transfers** (Property M4): Let $\mathbf{S}'$ be the population after a Pigou-Dalton transfer on $\mathbf{S} = \mathbf{G} \uplus \{s_1, s_2\}$ and choose the permutation matrices $\mathbf{P}_1$ and $\mathbf{P}_2$ according to Eq (63). Note that a Pigou-Dalton transfer does not affect the total or average indicator value $\overline{\mathbf{S}} = \overline{\mathbf{S}}'$.

$$\kappa(\mathbf{S})\ \mathbf{P}_1 = \frac{1}{|\mathbf{S}|} \begin{bmatrix} \begin{bmatrix} \begin{matrix} 1 \\ s/\overline{\mathbf{S}} \end{matrix} & : s \in \mathbf{G} \end{bmatrix} & \begin{matrix} 1 \\ s_1/\overline{\mathbf{S}} \end{matrix} & \begin{matrix} 1 \\ s_2/\overline{\mathbf{S}} \end{matrix} \end{bmatrix} \tag{63a}$$

$$\kappa(\mathbf{S}')\ \mathbf{P}_2 = \frac{1}{|\mathbf{S}|} \begin{bmatrix} \begin{bmatrix} \begin{matrix} 1 \\ s/\overline{\mathbf{S}} \end{matrix} & : s \in \mathbf{G} \end{bmatrix} & \begin{matrix} 1 \\ s_1'/\overline{\mathbf{S}} \end{matrix} & \begin{matrix} 1 \\ s_2'/\overline{\mathbf{S}} \end{matrix} \end{bmatrix} \tag{63b}$$

We can represent a Pigou-Dalton transfer with $p \in (0, 0.5]$ as multiplication by a double stochastic matrix, as shown in Eq (64), where $\mathbf{I}$ is an identity matrix.

$$\left[\begin{bmatrix} 1 \\ s/\bar{s} \end{bmatrix} : s \in \mathbf{G} \right] \begin{matrix} 1 & 1 \\ s'_1/\bar{s} & s'_2/\bar{s} \end{matrix} \Bigg] = \left[\left[\begin{bmatrix} 1 \\ s/\bar{s} \end{bmatrix} : s \in \mathbf{G} \right] \begin{matrix} 1 & 1 \\ s_1/\bar{s} & s_2/\bar{s} \end{matrix} \right] \begin{bmatrix} \mathbf{I} & 0 & 0 \\ 0 & (1-q) & q \\ 0 & q & (1-q) \end{bmatrix} \tag{64a}$$

$$\kappa(\mathbf{S}')\ \mathbf{P}_2 = \kappa(\mathbf{S})\ \mathbf{P}_1 \lambda \qquad \text{(where } \lambda \text{ is a double stochastic matrix)} \tag{64b}$$

Since stochastic matrices are closed under multiplication, any sequence of Pigou-Dalton transfers corresponds to a multiplication by some stochastic matrix λ.

Lemma 7. *If there exist a sequence of (non-empty) Pigou-Dalton transfers on population* $\mathbf{S}$ *to arrive at population* $\mathbf{S}'$*, then* $\langle \mathbf{S}' \rangle \sqsubset \langle \mathbf{S} \rangle$.

Proof. We obtain $Z_\kappa(\mathbf{S}') \subseteq Z_\kappa(\mathbf{S})$ from Eq (64) and Eq (17). We obtain $Z_\kappa(\mathbf{S}) \not\subseteq Z_\kappa(\mathbf{S}')$ since the inverse of the transfer matrix λ is not a valid stochastic matrix for $q \in (0, 0.5]$. It follows from Notation 4 and Definition 8 that $\langle \mathbf{S}' \rangle \sqsubset \langle \mathbf{S} \rangle$. □

5. **Bottom element** (Property M5):

Lemma 8. *The equivalence class of the bottom element* $\langle \bot_{\mathbf{S}} \rangle$ *contains all uniform distributions.*

Proof. The equivalence class of the bottom element is a predecessor for all other populations, as shown in Eq (65):

$$\kappa(\bot_{\mathbf{S}}) = \begin{bmatrix} 1 \\ 1 \end{bmatrix} = \kappa(\mathbf{S}) \begin{bmatrix} 1 \\ \vdots \\ 1 \end{bmatrix} \qquad \text{(by Definition 8)} \tag{65a}$$

$$\implies \langle \bot_{\mathbf{S}} \rangle \sqsubseteq \langle \mathbf{S} \rangle \qquad \text{(by Eq (16) and Definition 8)} \tag{65b}$$

Let $\mathbf{S}$ be an arbitrary uniform distribution, then its equivalence class is also a predecessor to the bottom element, as shown in Eq (66).

$$\kappa(\mathbf{S}) = \begin{bmatrix} 1 \\ 1 \end{bmatrix} \begin{bmatrix} 1/|\mathbf{S}| & \dots & 1/|\mathbf{S}| \end{bmatrix} \qquad \text{(by } \mathbf{S} \text{ being uniform)} \tag{66a}$$

$$= \kappa(\bot_{\mathbf{S}}) \begin{bmatrix} 1/|\mathbf{S}| & \dots & 1/|\mathbf{S}| \end{bmatrix} \qquad \text{(by Definition 8)} \tag{66b}$$

$$\implies \langle \mathbf{S} \rangle \sqsubseteq \langle \bot_{\mathbf{S}} \rangle \qquad \text{(by Eq (16) and Definition 8)} \tag{66c}$$

Eq (65) and Eq (66) imply $\langle \bot_{\mathbf{S}} \rangle = \langle \mathbf{S} \rangle$. □

Proofs for Section "From ordering to quantification":

Lemma 1: *Satisfying Property (weak $\star$) implies that the inequality measure satisfies the weak Property M1-M5.*

Proof.

- **Property M1**: Lemma 4 states that relabeling individuals/groups does not affect the equivalence class, and Eq (28a) ensures that all populations within an equivalence class obtain the same inequality index. This ensures Property M1.
- **Property M2**: Lemma 5 states that population duplication does not affect the equivalence class, and Eq (28a) ensures that all populations within an equivalence class obtain the same inequality index. This ensures Property M2.
- **Property M3**: Lemma 6 states that scaling the indicator variable does not affect the equivalence class, and Eq (28a) ensures that all populations within an equivalence class obtain the same inequality index. This ensures Property M3.
- **weak Property M4**: Lemma 7 states that the equivalence class after a Pigou-Dalton transfer is a predecessor of the original population. Eq (28a) ensures that predecessors obtain an inequality index that is less or equal. This ensures the *weak* Property M4.
- **Property M5**: Lemma 8 states that all uniform distributions are in the equivalence class of the bottom element, and Eq (28) ensures that all populations within this equivalence class obtain the inequality index zero. The non-negativity is then obtained from Eq (28a) since all populations are successors or equivalent to the bottom element. This ensures Property M5.

□

Lemma 2: *Satisfying Property (strict $\star$) implies that the inequality measure satisfies the strict Property M1-M5.*

Proof.

- **Property M1**: Lemma 4 states that relabeling individuals/groups does not affect the equivalence class, and Eq (29a) ensures that all populations within an equivalence class obtain the same inequality index. This ensures Property M1.
- **Property M2**: Lemma 5 states that population duplication does not affect the equivalence class, and Eq (29a) ensures that all populations within an equivalence class obtain the same inequality index. This ensures Property M2.

- **Property M3**: Lemma 6 states that scaling the indicator variable does not affect the equivalence class, and Eq (29a) ensures that all populations within an equivalence class obtain the same inequality index. This ensures Property M3.
- **strict Property M4**: Lemma 7 states that the equivalence class after a Pigou-Dalton transfer is a predecessor of the original population. Eq (29b) ensures that predecessors obtain a smaller inequality index. This ensures the *strict* Property M4.
- **Property M5**: Lemma 8 states that all uniform distributions are in the equivalence class of the bottom element, and Eq (29) ensures that all populations within this equivalence class obtain the inequality index zero. The non-negativity is then obtained from Eq (29b) since all populations are successors or equivalent to the bottom element. This ensures Property M5.

□
