# Supplementary material for: Quantifying redundancies and synergies with measures of inequality
Source: PLoS One. 2024 Nov 20;19(11):e0313281. doi: 10.1371/journal.pone.0313281 (PMC11578534; doi:10.1371/journal.pone.0313281)
Supplement: S2 Appendix — Proof of Theorem 1 (Properties of rf,p(v→) and If,p(S)).Proof of Lemma 3 (Minkowski addition to f-inequality addition).Proof of Theorem 2 (Pietra index and Generalized Entropy index are special cases of f -inequality). (PDF) [file pone.0313281.s002.pdf]

S2 Appendix. Properties and special cases of f-inequality.

Properties of f-inequality:

Theorem 1: *For a constant* $0 \leq p \leq 1$*:*

1. *the function* $r_{f,p}(\vec{v})$*:*
   (a) *quantifies any vector of slope one to zero:* $r_{f,p}\left(\left[\begin{smallmatrix}\ell\\ \ell\end{smallmatrix}\right]\right) = 0$
   (b) *quantifies the zero vector to zero:* $r_{f,p}\left(\left[\begin{smallmatrix}0\\ 0\end{smallmatrix}\right]\right) = 0$
   (c) *scales linearly in* $\vec{v}$ *where* $\ell \in \mathbb{R}$*:* $r_{f,p}(\ell\vec{v}) = \ell r_{f,p}(\vec{v})$
   (d) *is convex in* $\vec{v}$*:*
      - f*-inequality* $\ell \in \{0,1\}$*:* $r_{f,p}(\ell\vec{v}_1 + (1-\ell)\vec{v}_2) = \ell r_{f,p}(\vec{v}_1) + (1-\ell)r_{f,p}(\vec{v}_2)$
      - *weak* f*-inequality* $\ell \in (0,1)$*:* $r_{f,p}(\ell\vec{v}_1 + (1-\ell)\vec{v}_2) \leq \ell r_{f,p}(\vec{v}_1) + (1-\ell)r_{f,p}(\vec{v}_2)$
      - *strict* f*-inequality* $\ell \in (0,1)$*:* $r_{f,p}(\ell\vec{v}_1 + (1-\ell)\vec{v}_2) < \ell r_{f,p}(\vec{v}_1) + (1-\ell)r_{f,p}(\vec{v}_2)$
   (e) *satisfies a triangle inequality in* $\vec{v}$*:*
      - f*-inequality* $\mathrm{Slope}(\vec{v}_1) = \mathrm{Slope}(\vec{v}_2)$*:* $r_{f,p}(\vec{v}_1 + \vec{v}_2) = r_{f,p}(\vec{v}_1) + r_{f,p}(\vec{v}_2)$
      - *weak* f*-inequality* $\mathrm{Slope}(\vec{v}_1) \neq \mathrm{Slope}(\vec{v}_2)$*:* $r_{f,p}(\vec{v}_1 + \vec{v}_2) \leq r_{f,p}(\vec{v}_1) + r_{f,p}(\vec{v}_2)$
      - *strict* f*-inequality* $\mathrm{Slope}(\vec{v}_1) \neq \mathrm{Slope}(\vec{v}_2)$*:* $r_{f,p}(\vec{v}_1 + \vec{v}_2) < r_{f,p}(\vec{v}_1) + r_{f,p}(\vec{v}_2)$
2. *the function* $I_{f,p}(\mathbf{S})$*:*
   (a) *quantifies the bottom element to zero:* $I_{f,p}(\perp_{\mathbf{S}}) = 0$
   (b) *maintains the zonogon order:*
      - f*-inequality:* $\langle \mathbf{S}_1 \rangle = \langle \mathbf{S}_2 \rangle \implies I_{f,p}(\mathbf{S}_1) = I_{f,p}(\mathbf{S}_2)$
      - *weak* f*-inequality:* $\langle \mathbf{S}_1 \rangle \sqsubseteq \langle \mathbf{S}_2 \rangle \implies I_{f,p}(\mathbf{S}_1) \leq I_{f,p}(\mathbf{S}_2)$
      - *strict* f*-inequality:* $\langle \mathbf{S}_1 \rangle \sqsubset \langle \mathbf{S}_2 \rangle \implies I_{f,p}(\mathbf{S}_1) < I_{f,p}(\mathbf{S}_2)$

Proof.

1. Properties of $r_{f,p}(\vec{v})$:
   (a) Non-zero vectors of slope one $\vec{v} = \left[\begin{smallmatrix}\ell\\ \ell\end{smallmatrix}\right]$:

$$r_{f,p}\left(\left[\begin{smallmatrix}\ell\\ \ell\end{smallmatrix}\right]\right) = (p\ell + (1-p)\ell) \cdot f\left(\frac{\ell}{p\ell+(1-p)\ell}\right) = \ell \cdot f\left(\frac{\ell}{\ell}\right) \quad \text{(by Definition 12)} \tag{67a}$$

$$= \ell \cdot f(1) = 0 \quad \text{(by Notation 6)} \tag{67b}$$

(b) The zero vector $\vec{v} = \left[\begin{smallmatrix}0\\0\end{smallmatrix}\right]$:

$$r_{f,p}\left(\left[\begin{smallmatrix}0\\0\end{smallmatrix}\right]\right) = 0 \cdot f\left(\tfrac{0}{0}\right) \qquad \text{(by Eq (67a))} \qquad (68a)$$

$$= 0 \qquad \text{(by Notation 6)} \qquad (68b)$$

(c) Linear scaling of vectors:

$$r_{f,p}(\ell\vec{v}) = r_{f,p}\left(\ell\left[\begin{smallmatrix}x\\y\end{smallmatrix}\right]\right) = (p\ell x + (1-p)\ell y) \cdot f\left(\tfrac{\ell x}{p\ell x+(1-p)\ell y}\right) \qquad \text{(by Definition 12)} \qquad (69a)$$

$$= \ell\,(px + (1-p)y) \cdot f\left(\tfrac{x}{px+(1-p)y}\right) \qquad (69b)$$

$$= \ell r_{f,p}\left(\left[\begin{smallmatrix}x\\y\end{smallmatrix}\right]\right) = \ell r_{f,p}(\vec{v}) \qquad (69c)$$

(d) Convexity in $\vec{v}$:

- Assume $\ell = \{0,1\}$, then Eq (70) simplifies into a simple identity:

$$r_{f,p}(\ell\vec{v}_1 + (1-\ell)\vec{v}_2) = \ell r_{f,p}(\vec{v}_1) + (1-\ell)r_{f,p}(\vec{v}_2) \qquad (70)$$

- Assume $\ell \in (0,1)$: We use the following definitions as abbreviation:

$$\vec{v}_1 := \left[\begin{smallmatrix}x_1\\y_1\end{smallmatrix}\right] \qquad a_1 := x_1p + y_1(1-p) \qquad b_1 := \tfrac{\ell a_1}{\ell a_1+(1-\ell)a_2}$$
$$\vec{v}_2 := \left[\begin{smallmatrix}x_2\\y_2\end{smallmatrix}\right] \qquad a_2 := x_2p + y_2(1-p) \qquad b_2 := \tfrac{(1-\ell)a_2}{\ell a_1+(1-\ell)a_2}$$

The cases of $a_1 = 0$ and $a_2 = 0$ are covered by the convention $0f(\tfrac{0}{0}) = 0$ (Notation 6). Therefore, we can assume they are non-zero and utilize the following two relations: $0 < b_1 < 1$ and $b_2 = 1 - b_1$. If f is strictly convex, let $(\sim) = (<)$, otherwise let $(\sim) = (\leq)$.

$$r_{f,p}(\ell\vec{v}_1 + (1-\ell)\vec{v}_2) = (\ell a_1 + (1-\ell)a_2) \cdot f\left(\frac{\ell x_1 + (1-\ell)x_2}{\ell a_1 + (1-\ell)a_2}\right) \qquad \text{(by Definition 12)} \qquad (71a)$$

$$= (\ell a_1 + (1-\ell)a_2) \cdot f\left(b_1\frac{x_1}{a_1} + b_2\frac{x_2}{a_2}\right) \qquad (71b)$$

$$\sim (\ell a_1 + (1-\ell)a_2) \cdot \left(b_1 f\left(\frac{x_1}{a_1}\right) + b_2 f\left(\frac{x_2}{a_2}\right)\right) \qquad \text{(by convexity of } f\text{)} \qquad (71c)$$

$$= \ell a_1 f\left(\frac{x_1}{a_1}\right) + (1-\ell)a_2 f\left(\frac{x_2}{a_2}\right) \qquad (71d)$$

$$= \ell r_{f,p}(\vec{v}_1) + (1-\ell)r_{f,p}(\vec{v}_2) \qquad (71e)$$

(e) Triangle inequality in $\vec{v}$:

- Assume $\mathrm{Slope}(\vec{v}_1) = \mathrm{Slope}(\vec{v}_2)$: Then there exists an $\ell \in \mathbb{R}$ such that $\ell\vec{v}_1 = \vec{v}_2$.

$$\begin{aligned}
r_{f,p}(\vec{v}_1 + \vec{v}_2) = r_{f,p}(\vec{v}_1 + \ell\vec{v}_1) &= r_{f,p}((1+\ell)\vec{v}_1) && (72a)\\
&= (1+\ell) r_{f,p}(\vec{v}_1) \quad \text{(by Theorem 1 nr. 1.c)} && (72b)\\
&= r_{f,p}(\vec{v}_1) + \ell r_{f,p}(\vec{v}_1) && (72c)\\
&= r_{f,p}(\vec{v}_1) + r_{f,p}(\ell\vec{v}_1) \quad \text{(by Theorem 1 nr. 1.c)} && (72d)\\
&= r_{f,p}(\vec{v}_1) + r_{f,p}(\vec{v}_2) && (72e)
\end{aligned}$$

- Assume $\mathrm{Slope}(\vec{v}_1) \neq \mathrm{Slope}(\vec{v}_2)$:

  If f is strictly convex, let $(\sim) = (<)$, otherwise let $(\sim) = (\leq)$.

$$\begin{aligned}
r_{f,p}(\ell\vec{v}_1 + (1-\ell)\vec{v}_2) &\sim \ell r_{f,p}(\vec{v}_1) + (1-\ell) r_{f,p}(\vec{v}_2) \quad \text{(by Theorem 1 nr. 1.d)} && (73a)\\
r_{f,p}(0.5(\vec{v}_1 + \vec{v}_2)) &\sim 0.5 r_{f,p}(\vec{v}_1) + 0.5 r_{f,p}(\vec{v}_2) \quad \text{(let } \ell = 0.5\text{)} && (73b)\\
0.5 r_{f,p}(\vec{v}_1 + \vec{v}_2) &\sim 0.5 r_{f,p}(\vec{v}_1) + 0.5 r_{f,p}(\vec{v}_2) \quad \text{(by Theorem 1 nr. 1.c)} && (73c)\\
r_{f,p}(\vec{v}_1 + \vec{v}_2) &\sim r_{f,p}(\vec{v}_1) + r_{f,p}(\vec{v}_2) && (73d)
\end{aligned}$$

2. Properties of $I_{f,p}(\mathbf{S})$:

   (a) Bottom element $\bot_{\mathbf{S}} = \{1\}$:

$$I_{f,p}(\bot_{\mathbf{S}}) = \sum_{\vec{v} \in \kappa(\{1\})} r_{f,p}(\vec{v}) = r_{f,p}\left(\left[\begin{smallmatrix}1\\1\end{smallmatrix}\right]\right) = 0 \quad \text{(by Theorem 1 nr. 1.a)} \qquad (74)$$

   (b) Zonogon order:

   - Assume $Z_\kappa(\mathbf{S}_1) = Z_\kappa(\mathbf{S}_2)$, which equals $\langle \mathbf{S}_1 \rangle = \langle \mathbf{S}_2 \rangle$ by Definition 7 and Notation 4: In this case, both zonogons have the same boundary. Since the boundary consists of the generating vectors sorted by slope, the generating vectors of identical slope have the same sum:

$$\begin{aligned}
\forall x \in \mathbb{R} : &\quad \sum_{\substack{\vec{v} \in \kappa(\mathbf{S}_1)\\ \mathrm{Slope}(\vec{v}) = x}} \vec{v} = \sum_{\substack{\vec{v} \in \kappa(\mathbf{S}_2)\\ \mathrm{Slope}(\vec{v}) = x}} \vec{v} \quad \text{(by } Z_\kappa(\mathbf{S}_1) = Z_\kappa(\mathbf{S}_2)\text{)} && (75a)\\
\forall x \in \mathbb{R} : &\quad r_{f,p}\left(\sum_{\substack{\vec{v} \in \kappa(\mathbf{S}_1)\\ \mathrm{Slope}(\vec{v}) = x}} \vec{v} \right) = r_{f,p}\left(\sum_{\substack{\vec{v} \in \kappa(\mathbf{S}_2)\\ \mathrm{Slope}(\vec{v}) = x}} \vec{v} \right) && (75b)\\
\forall x \in \mathbb{R} : &\quad \sum_{\substack{\vec{v} \in \kappa(\mathbf{S}_1)\\ \mathrm{Slope}(\vec{v}) = x}} r_{f,p}(\vec{v}) = \sum_{\substack{\vec{v} \in \kappa(\mathbf{S}_2)\\ \mathrm{Slope}(\vec{v}) = x}} r_{f,p}(\vec{v}) \quad \text{(by Theorem 1 nr. 1.e)} && (75c)
\end{aligned}$$

$$\sum_{x\in\mathbb{R}} \sum_{\substack{\vec{v}\in\kappa(\mathbf{S}_1)\\ \text{Slope}(\vec{v})=x}} r_{f,p}(\vec{v}) = \sum_{x\in\mathbb{R}} \sum_{\substack{\vec{v}\in\kappa(\mathbf{S}_2)\\ \text{Slope}(\vec{v})=x}} r_{f,p}(\vec{v}) \tag{75d}$$

$$\sum_{\vec{v}\in\kappa(\mathbf{S}_1)} r_{f,p}(\vec{v}) = \sum_{\vec{v}\in\kappa(\mathbf{S}_2)} r_{f,p}(\vec{v}) \tag{75e}$$

$$I_{f,p}(\mathbf{S}_1) = I_{f,p}(\mathbf{S}_2) \tag{75f}$$

- Assume $Z_\kappa(\mathbf{S}_1) \subset Z_\kappa(\mathbf{S}_2)$, which equals $\langle \mathbf{S}_1 \rangle \sqsubset \langle \mathbf{S}_2 \rangle$ by Definition 7 and Definition 8: In this case, there exists a stochastic matrix λ which combines some vectors from κ_2 with different slope:

$$\kappa_1 := \kappa(\mathbf{S}_1) \qquad \kappa_2 := \kappa(\mathbf{S}_2) \qquad \kappa_1 = \kappa_2\lambda \tag{76}$$

Let κ_1 be a $2 \times a$ stochastic matrix, κ_2 be a $2 \times b$ stochastic matrix and λ be a $b \times a$ stochastic matrix. We write $\kappa_2[:,i]$ to refer to the ith column of matrix κ_2 and write $\lambda[i,j]$ for the element at row $i \in \{1,..,b\}$ and column $j \in \{1,..,a\}$. Since λ is a stochastic matrix, its rows sum to one $\forall i \in \{1,..,b\} \, : \, \sum_{j=1}^{a} \lambda[i,j] = 1$. If f is strictly convex, let $(\sim) = (<)$, otherwise let $(\sim) = (\leq)$.

$$\begin{aligned}
I_{f,p}(\mathbf{S}_1) &= \sum_{j=1}^{a} r_{f,p}(\kappa_1[:,j]) && \text{(by Definition 12)} && (77a)\\
&= \sum_{j=1}^{a} r_{f,p}\Big(\sum_{i=1}^{b} \kappa_2[:,i]\lambda[i,j]\Big) && (\text{by } \kappa_1 = \kappa_2\lambda) && (77b)\\
&\sim \sum_{j=1}^{a}\sum_{i=1}^{b} r_{f,p}(\kappa_2[:,i]\lambda[i,j]) && \text{(by Theorem 1 nr. 1.e)} && (77c)\\
&= \sum_{j=1}^{a}\sum_{i=1}^{b} \lambda[i,j] r_{f,p}(\kappa_2[:,i]) && \text{(by Theorem 1 nr. 1.c)} && (77d)\\
&= \sum_{i=1}^{b} r_{f,p}(\kappa_2[:,i]) && \Big(\text{by } \sum_{j=1}^{a}\lambda[i,j] = 1\Big) && (77e)\\
&= I_{f,p}(\mathbf{S}_2) && \text{(by Definition 12)} && (77f)
\end{aligned}$$

□

Additivity of f-inequality:

Proof of Lemma 3 from Section "Defining f-inequality":

Consider two non-empty sets of populations with equal cardinality ($|\mathbf{A}| = |\mathbf{B}|$), then:

$$\textit{f-inequality:} \qquad \sum_{\mathbf{S}\in\mathbf{A}} Z_\kappa(\mathbf{S}) = \sum_{\mathbf{S}\in\mathbf{B}} Z_\kappa(\mathbf{S}) \implies \sum_{\mathbf{S}\in\mathbf{A}} I_{f,p}(\mathbf{S}) = \sum_{\mathbf{S}\in\mathbf{B}} I_{f,p}(\mathbf{S}) \tag{78a}$$

$$\textit{weak f-inequality:} \qquad \sum_{\mathbf{S}\in\mathbf{A}} Z_\kappa(\mathbf{S}) \subseteq \sum_{\mathbf{S}\in\mathbf{B}} Z_\kappa(\mathbf{S}) \implies \sum_{\mathbf{S}\in\mathbf{A}} I_{f,p}(\mathbf{S}) \leq \sum_{\mathbf{S}\in\mathbf{B}} I_{f,p}(\mathbf{S}) \tag{78b}$$

$$\textit{strict f-inequality:} \qquad \sum_{\mathbf{S}\in\mathbf{A}} Z_\kappa(\mathbf{S}) \subset \sum_{\mathbf{S}\in\mathbf{B}} Z_\kappa(\mathbf{S}) \implies \sum_{\mathbf{S}\in\mathbf{A}} I_{f,p}(\mathbf{S}) < \sum_{\mathbf{S}\in\mathbf{B}} I_{f,p}(\mathbf{S}) \tag{78c}$$

Proof. Let $m = |\mathbf{A}| = |\mathbf{B}|$ and $(\sim,\approx) \in \{(=,=),(\subseteq,\leq),(\subset,<)\}$. We use the notation $\mathbf{A}[i]$ and $\mathbf{B}[i]$ with $1 \leq i \leq m$ to indicate a specific population within the set $\mathbf{A}$ and $\mathbf{B}$ respectively.

$$\begin{aligned}
\sum_{i=1}^{m} Z_\kappa(\mathbf{A}[i]) &\sim \sum_{i=1}^{m} Z_\kappa(\mathbf{B}[i]) \\
Z\left(\begin{bmatrix}\kappa(\mathbf{A}[1]) & \dots & \kappa(\mathbf{A}[m])\end{bmatrix}\right) &\sim Z\left(\begin{bmatrix}\kappa(\mathbf{B}[1]) & \dots & \kappa(\mathbf{B}[m])\end{bmatrix}\right) && \text{(by Definition 9)} \\
Z\left(\frac{1}{m}\begin{bmatrix}\kappa(\mathbf{A}[1]) & \dots & \kappa(\mathbf{A}[m])\end{bmatrix}\right) &\sim Z\left(\frac{1}{m}\begin{bmatrix}\kappa(\mathbf{B}[1]) & \dots & \kappa(\mathbf{B}[m])\end{bmatrix}\right) && \text{(scale zonogon to (1,1))} \\
\sum_{\vec{v}\in\frac{1}{m}[\kappa(\mathbf{A}[1]) \dots \kappa(\mathbf{A}[m])]} r_{f,p}(\vec{v}) &\approx \sum_{\vec{v}\in\frac{1}{m}[\kappa(\mathbf{B}[1]) \dots \kappa(\mathbf{B}[m])]} r_{f,p}(\vec{v}) && \text{(by Def. 8, Thm. 1 nr. 2.b, Def. 12)} \\
\sum_{\vec{v}\in[\kappa(\mathbf{A}[1]) \dots \kappa(\mathbf{A}[m])]} \frac{1}{m} r_{f,p}(\vec{v}) &\approx \sum_{\vec{v}\in[\kappa(\mathbf{B}[1]) \dots \kappa(\mathbf{B}[m])]} \frac{1}{m} r_{f,p}(\vec{v}) && \text{(by Theorem 1 nr. 1.c)} \\
\sum_{i=1}^{m} \sum_{\vec{v}\in\kappa(\mathbf{A}[i])} r_{f,p}(\vec{v}) &\approx \sum_{i=1}^{m} \sum_{\vec{v}\in\kappa(\mathbf{B}[i])} r_{f,p}(\vec{v}) && \text{(multiply } m\text{, split sum)} \\
\sum_{i=1}^{m} I_{f,p}(\mathbf{A}[i]) &\approx \sum_{i=1}^{m} I_{f,p}(\mathbf{B}[i]) && \text{(by Definition 12)} \\
\sum_{\mathbf{S}\in\mathbf{A}} I_{f,p}(\mathbf{S}) &\approx \sum_{\mathbf{S}\in\mathbf{B}} I_{f,p}(\mathbf{S}) && \text{(change notation)}
\end{aligned}$$

□

Special cases of f-inequality:

Proof of Theorem 2 from Section "Defining f-inequality":

The Pietra index and Generalized Entropy index are special cases of f-inequality:

$$R(\mathbf{S}) = I_{f,p}(\mathbf{S}) \qquad \textit{where: } p = 0 \textit{ and } f(t) = \frac{|t-1|}{2} \tag{79a}$$

$$GE_c(\mathbf{S}) = I_{f,p}(\mathbf{S}) \qquad \textit{where: } p = 0 \textit{ and } f(t) = \frac{t^{1-c} - t}{c(c-1)} \tag{79b}$$

$$GE_1(\mathbf{S}) = I_{f,p}(\mathbf{S}) \qquad \textit{where: } p = 0 \textit{ and } f(t) = -\ln(t) \tag{79c}$$

$$GE_0(\mathbf{S}) = I_{f,p}(\mathbf{S}) \qquad \textit{where: } p = 0 \textit{ and } f(t) = t\ln(t) \tag{79d}$$

Proof. We can simplify the generalized inequality function for $p = 0$ as shown in Eq (80):

$$I_{f,0}(\mathbf{S}) = \frac{1}{|\mathbf{S}|}\sum_{s\in\mathbf{S}} \frac{s}{\overline{\mathbf{S}}} \cdot f\left(\frac{\overline{\mathbf{S}}}{s}\right) \tag{80}$$

- Pietra index:

$$\begin{aligned}
I_{f,0}(\mathbf{S}) &= \frac{1}{|\mathbf{S}|}\sum_{s\in\mathbf{S}} \frac{s}{\overline{\mathbf{S}}} \cdot f\left(\frac{\overline{\mathbf{S}}}{s}\right) \\
&= \frac{1}{|\mathbf{S}|}\sum_{s\in\mathbf{S}} \frac{s}{\overline{\mathbf{S}}} \cdot \frac{|\frac{\overline{\mathbf{S}}}{s} - 1|}{2} && \text{using: } f(t) = \frac{|t-1|}{2} \\
&= \frac{1}{2|\mathbf{S}|}\sum_{s\in\mathbf{S}} \frac{|\overline{\mathbf{S}} - s|}{\overline{\mathbf{S}}} && \text{using: } s \geq 0 \\
&= \frac{1}{2|\mathbf{S}|}\sum_{s\in\mathbf{S}} \frac{|s - \overline{\mathbf{S}}|}{\overline{\mathbf{S}}} && \text{using: } |a-b| = |b-a| \\
&= R(\mathbf{S}) && \text{(by Eq (10))}
\end{aligned} \tag{81}$$

The function $f(t) = \frac{|t-1|}{2}$ is a well known generator function for an f-divergences from the total variation distance.

- $\mathrm{GE}_c(\mathbf{S})$ index with $c \notin \{0,1\}$:

$$\begin{aligned}
I_{f,0}(\mathbf{S}) &= \frac{1}{|\mathbf{S}|}\sum_{s\in\mathbf{S}} \frac{s}{\overline{\mathbf{S}}} \cdot f\left(\frac{\overline{\mathbf{S}}}{s}\right) \\
&= \frac{1}{|\mathbf{S}|}\sum_{s\in\mathbf{S}} \frac{s}{\overline{\mathbf{S}}} \cdot \frac{\left(\frac{\overline{\mathbf{S}}}{s}\right)^{1-c} - \frac{\overline{\mathbf{S}}}{s}}{c(c-1)} && \text{using: } \frac{t^{1-c} - t}{c(c-1)} \\
&= \frac{1}{|\mathbf{S}|}\sum_{s\in\mathbf{S}} \frac{s}{\overline{\mathbf{S}}} \cdot \frac{\frac{\overline{\mathbf{S}}}{s} \cdot \left(\frac{s}{\overline{\mathbf{S}}}\right)^{c} - \frac{\overline{\mathbf{S}}}{s}}{c(c-1)} \\
&= \frac{1}{c(c-1)}\frac{1}{|\mathbf{S}|}\sum_{s\in\mathbf{S}} \left(\left(\frac{s}{\overline{\mathbf{S}}}\right)^{c} - 1\right) \\
&= \mathrm{GE}_c(\mathbf{S}) && \text{(by Eq (11))}
\end{aligned} \tag{82}$$

The function $f(t)$ satisfies the requirements for a generator function of an f-divergence:

1. $f(1) = \frac{1}{c(c-1)}(1-1) = 0$.
2. $f(t)$ is convex for $t > 0$ and $c \in \mathbb{R} \setminus \{0,1\}$ since $f''(t) = \left(\frac{1}{t}\right)^{c+1} \geq 0$
3. $f(t)$ is finite for $t > 0$ and $c \in \mathbb{R} \setminus \{0,1\}$.

- $\mathrm{GE}_1(\mathbf{S})$ index with $c = 1$ (Theil index):

$$\begin{aligned}
I_{f,0}(\mathbf{S}) &= \frac{1}{|\mathbf{S}|}\sum_{s\in\mathbf{S}} \frac{s}{\overline{\overline{\mathbf{S}}}} \cdot f\left(\frac{\overline{\mathbf{S}}}{s}\right) \\
&= \frac{1}{|\mathbf{S}|}\sum_{s\in\mathbf{S}} \frac{s}{\overline{\overline{\mathbf{S}}}} \cdot \left(-\ln\left(\frac{\overline{\mathbf{S}}}{s}\right)\right) \quad \text{using: } f(t) = -\ln(t) \\
&= \frac{1}{|\mathbf{S}|}\sum_{s\in\mathbf{S}} \frac{s}{\overline{\overline{\mathbf{S}}}} \cdot \ln\left(\frac{s}{\overline{\overline{\mathbf{S}}}}\right) \\
&= \mathrm{GE}_1(\mathbf{S}) \qquad \text{(by Eq (11))}
\end{aligned} \tag{83}$$

The function $f(t) = -\ln(t)$ is a well known generator function for an f-divergences from the reverse Kullback–Leibler divergence.

- $\mathrm{GE}_0(\mathbf{S})$ index with $c = 0$:

$$\begin{aligned}
I_{f,0}(\mathbf{S}) &= \frac{1}{|\mathbf{S}|}\sum_{s\in\mathbf{S}} \frac{s}{\overline{\overline{\mathbf{S}}}} \cdot f\left(\frac{\overline{\mathbf{S}}}{s}\right) \\
&= \frac{1}{|\mathbf{S}|}\sum_{s\in\mathbf{S}} \frac{s}{\overline{\overline{\mathbf{S}}}} \cdot \frac{\overline{\mathbf{S}}}{s}\ln\left(\frac{\overline{\mathbf{S}}}{s}\right) \quad \text{using: } f(t) = t\ln(t) \\
&= -\frac{1}{|\mathbf{S}|}\sum_{s\in\mathbf{S}} \ln\left(\frac{s}{\overline{\overline{\mathbf{S}}}}\right) \\
&= \mathrm{GE}_0(\mathbf{S}) \qquad \text{(by Eq (11))}
\end{aligned} \tag{84}$$

The function $f(t) = t\ln(t)$ is a well known generator function for an f-divergences from the Kullback–Leibler divergence.

□
