# Supplementary material for: Quantifying redundancies and synergies with measures of inequality
Source: PLoS One. 2024 Nov 20;19(11):e0313281. doi: 10.1371/journal.pone.0313281 (PMC11578534; doi:10.1371/journal.pone.0313281)
Supplement: S3 Appendix — Proof of Theorem 3 (the constructed decomposition satisfies Property U1-U4). (PDF) [file pone.0313281.s003.pdf]

**S3 Appendix. Decomposition properties.**

**Proof of Theorem 3 from Section "Decomposing f-inequality":** *Definition 17 satisfies Property U1-U4.*

*Proof.*

- **Property U1** (commutativity): The join operator (convex hull of zonogons) is invariant to the order of zonogons. Therefore, the measure $I^{\cup}_{f,p}(\cdot)$ of Definition 17 is invariant to the order of attribute sets in an atom:

$$\forall \alpha \in \mathcal{A}(n): \quad \bigsqcup_{\mathbf{a}\in\alpha} \langle \Gamma(\mathbf{a},\mathbf{M})\rangle = \bigsqcup_{\mathbf{a}\in\{\sigma(\mathbf{a})\,:\,\mathbf{a}\in\alpha\}} \langle \Gamma(\mathbf{a},\mathbf{M})\rangle \qquad \text{(commutativity of lattice join)}$$

$$I^{\cup}_{f,p}(\alpha,\mathbf{M}) = I^{\cup}_{f,p}(\{\sigma(\mathbf{a}) \;:\; \mathbf{a}\in\alpha\},\mathbf{M}) \qquad \text{(by Theorem 1 nr. 2.b)}$$

- **Property U2** (monotonicity): The join element is monotonically increasing and thus also the measure $I^{\cup}_{f,p}(\cdot)$ of Definition 17.

$$\forall \alpha \in \mathcal{A}(n),\; \forall \mathbf{a} \in \mathcal{P}(\{1,..,n\}):$$

$$\bigsqcup_{\mathbf{b}\in\alpha} \langle \Gamma(\mathbf{b},\mathbf{M})\rangle \sqsubseteq \left(\bigsqcup_{\mathbf{b}\in\alpha} \langle \Gamma(\mathbf{b},\mathbf{M})\rangle\right) \sqcup \langle \Gamma(\mathbf{a},\mathbf{M})\rangle \qquad \text{(monotonicity of lattice join)}$$

$$I^{\cup}_{f,p}(\alpha,\mathbf{M}) \leq I^{\cup}_{f,p}(\alpha \cup \{\mathbf{a}\},\mathbf{M}) \qquad \text{(by Theorem 1 nr. 2.b)}$$

- **Property U3** (self-inequality): The join of a single population is an identity such that the measure $I^{\cup}_{f,p}(\cdot)$ of Definition 17 equals $I_{f,p}(\cdot)$.

$$\forall \mathbf{a} \in \mathcal{P}(\{1,..,n\}): \; I^{\cup}_{f,p}(\{\mathbf{a}\},\mathbf{M}) = I_{f,p}\left(\bigsqcup_{\mathbf{b}\in\{\mathbf{a}\}} \langle \Gamma(\mathbf{b},\mathbf{M})\rangle\right) = I_{f,p}\left(\Gamma(\mathbf{a},\mathbf{M})\right) \qquad \text{(by Definition 17)}$$

- **Property U4** (non-negativity): We begin with the required preliminaries:
  - The join on the union lattice can be expressed as shown in Eq (85a) [1], using the function 'reduce' of Eq (46).
  - The function reduce$(\subset,\cdot)$ does not affect the convex hull of the underlying zonogons, which provides Eq (85b) from Eq (85a).
  - From Eq (85b), we obtain the monotonicity of the cumulative measure on the lattice shown in Eq (85c).
  - We notate the set of immediate successors of $\alpha$ as $\alpha^{+}$ (Eq (85d)).
  - The Möbius inverse can be computed using an inclusion-exclusion relation as shown in Eq (85e) [1–3].

– Since the inclusion-exclusion of a constant is the constant itself, we obtain Eq (85f) from Eq (85e).

– Using Eq (85f) and Definition 16 (Eq (40b)) provides Eq (85g).

$$\alpha \curlyvee \beta = \text{reduce}(\subset, \alpha \cup \beta) \tag{85a}$$

$$\bigsqcup_{\mathbf{c}\in(\alpha\curlyvee\beta)} \langle \Gamma(\mathbf{c},\mathbf{M})\rangle = \bigsqcup_{\mathbf{c}\in(\alpha\cup\beta)} \langle \Gamma(\mathbf{c},\mathbf{M})\rangle \tag{85b}$$

$$\alpha \preceq \beta \Longrightarrow I^{\cup}_{f,p}(\alpha,\mathbf{M}) \leq I^{\cup}_{f,p}(\beta,\mathbf{M}) \tag{85c}$$

$$\alpha^{+} := \{\beta \in \mathcal{A}(n) \, : \, \alpha \prec \beta \text{ and } \neg(\exists\gamma \in \mathcal{A}(n))[\alpha \prec \gamma \text{ and } \gamma \prec \beta]\} \tag{85d}$$

$$\alpha \neq \top_{\cup} : \qquad \sum_{\beta\in\dagger\alpha} I^{\delta}_{f,p}(\beta,\mathbf{M}) = \sum_{\emptyset\neq\mathbf{B}\subseteq\alpha^{+}} (-1)^{|\mathbf{B}|-1}\left( I^{\cup}_{f,p}(\top_{\cup},\mathbf{M}) - I^{\cup}_{f,p}(\bigcurlyvee_{\beta\in\mathbf{B}} \beta,\mathbf{M})\right) \tag{85e}$$

$$\alpha \neq \top_{\cup} : \qquad \sum_{\beta\in\dagger\alpha} I^{\delta}_{f,p}(\beta,\mathbf{M}) = I^{\cup}_{f,p}(\top_{\cup},\mathbf{M}) - \sum_{\emptyset\neq\mathbf{B}\subseteq\alpha^{+}} (-1)^{|\mathbf{B}|-1} I^{\cup}_{f,p}(\bigcurlyvee_{\beta\in\mathbf{B}} \beta,\mathbf{M}) \tag{85f}$$

$$\alpha \neq \top_{\cup} : \qquad I^{\delta}_{f,p}(\alpha,\mathbf{M}) = -I^{\cup}_{f,p}(\alpha,\mathbf{M}) + \sum_{\emptyset\neq\mathbf{B}\subseteq\alpha^{+}} (-1)^{|\mathbf{B}|-1} I^{\cup}_{f,p}(\bigcurlyvee_{\beta\in\mathbf{B}} \beta,\mathbf{M}) \tag{85g}$$

To show the non-negativity of partial contributions, we split the case of $\alpha = \top_{\cup}$:

1. Assume $\alpha = \top_{\cup} = \{\{1,..,n\}\}$: In this case, its strict upset is the empty set ($\dagger\alpha = \{\}$). We obtain from Definition 16 that its partial contribution is zero and thus non-negative:

$$\alpha = \top_{\cup} : \qquad I^{\delta}_{f,p}(\alpha,\mathbf{M}) = I^{\cup}_{f,p}(\top_{\cup},\mathbf{M}) - I^{\cup}_{f,p}(\alpha,\mathbf{M}) = 0 \geq 0 \tag{86}$$

2. Assume $\alpha \neq \top_{\cup}$: In this case, the strict upset of $\alpha$ is non-empty. To show the non-negativity, we construct a lower and upper bound for the two components of Eq (85g). First, we obtain the lower bound on the inclusion-exclusion relation from Corollary 2 as shown in Eq (87) and simplify it using Eq (85b).

$$\alpha \neq \top_{\cup} : \qquad I_{f,p}\left(\bigsqcap_{\gamma\in\alpha^{+}} \bigsqcup_{\mathbf{c}\in\gamma} \langle \Gamma(\mathbf{c},\mathbf{M})\rangle\right) \leq \sum_{\emptyset\neq\mathbf{C}\subseteq\alpha^{+}} (-1)^{|\mathbf{C}|-1} I_{f,p}\left(\bigsqcup_{\gamma\in\mathbf{C}} \bigsqcup_{\mathbf{c}\in\gamma} \langle \Gamma(\mathbf{c},\mathbf{M})\rangle\right) \tag{87a}$$

$$\alpha \neq \top_{\cup} : \qquad I_{f,p}\left(\bigsqcap_{\gamma\in\alpha^{+}} \bigsqcup_{\mathbf{c}\in\gamma} \langle \Gamma(\mathbf{c},\mathbf{M})\rangle\right) \leq \sum_{\emptyset\neq\mathbf{C}\subseteq\alpha^{+}} (-1)^{|\mathbf{C}|-1} I_{f,p}\left(\bigsqcup_{\mathbf{c}\in\left(\bigcup_{\gamma\in\mathbf{C}}(\gamma)\right)} \langle \Gamma(\mathbf{c},\mathbf{M})\rangle\right) \tag{87b}$$

$$\alpha \neq \top_{\cup} : \qquad I_{f,p}\left(\bigsqcap_{\gamma\in\alpha^{+}} \bigsqcup_{\mathbf{c}\in\gamma} \langle \Gamma(\mathbf{c},\mathbf{M})\rangle\right) \leq \sum_{\emptyset\neq\mathbf{C}\subseteq\alpha^{+}} (-1)^{|\mathbf{C}|-1} I^{\cup}_{f,p}(\bigcurlyvee_{\gamma\in\mathbf{C}} \gamma,\mathbf{M}) \tag{87c}$$

Second, we obtain an upper bound for atom $\alpha$ based on its immediate successors as shown in

Eq (88) from Eq (85c).

$$\alpha \neq \top_{\cup}, \forall \gamma \in \alpha^{+}: \qquad \bigsqcup_{\mathbf{a}\in\alpha} \langle \Gamma(\mathbf{a},\mathbf{M}) \rangle \sqsubseteq \bigsqcup_{\mathbf{c}\in\gamma} \langle \Gamma(\mathbf{c},\mathbf{M}) \rangle \tag{88a}$$

$$\alpha \neq \top_{\cup}: \qquad \bigsqcup_{\mathbf{a}\in\alpha} \langle \Gamma(\mathbf{a},\mathbf{M}) \rangle \sqsubseteq \bigsqcap_{\gamma\in\alpha^{+}} \bigsqcup_{\mathbf{c}\in\gamma} \langle \Gamma(\mathbf{c},\mathbf{M}) \rangle \tag{88b}$$

$$\alpha \neq \top_{\cup}: \qquad I_{f,p}\left( \bigsqcup_{\mathbf{a}\in\alpha} \langle \Gamma(\mathbf{a},\mathbf{M}) \rangle \right) \leq I_{f,p}\left( \bigsqcap_{\gamma\in\alpha^{+}} \bigsqcup_{\mathbf{c}\in\gamma} \langle \Gamma(\mathbf{c},\mathbf{M}) \rangle \right) \tag{88c}$$

By transitivity, we obtain Eq (89a) from Eq (87c) and Eq (88c). Re-arranging both terms demonstrates the desired non-negativity of the partial contributions.

$$\alpha \neq \top_{\cup}: \qquad I^{\cup}_{f,p}(\alpha,\mathbf{M}) \leq \sum_{\emptyset\neq\mathbf{C}\subseteq\alpha^{+}} (-1)^{|\mathbf{C}|-1} I^{\cup}_{f,p}(\bigcurlyvee_{\gamma\in\mathbf{C}} \gamma,\mathbf{M}) \tag{89a}$$

$$\alpha \neq \top_{\cup}: \qquad 0 \leq -I^{\cup}_{f,p}(\alpha,\mathbf{M}) + \sum_{\emptyset\neq\mathbf{C}\subseteq\alpha^{+}} (-1)^{|\mathbf{C}|-1} I^{\cup}_{f,p}(\bigcurlyvee_{\gamma\in\mathbf{C}} \gamma,\mathbf{M}) \tag{89b}$$

$$\alpha \neq \top_{\cup}: \qquad 0 \leq I^{\delta}_{f,p}(\alpha,\mathbf{M}) \qquad \text{(applying Eq (85g))} \tag{89c}$$

From Eq (86) and Eq (89c) we obtain the non-negativity of the decomposition and thus Property U4.

$$\forall \alpha \in \mathcal{A}(n): \quad 0 \leq I^{\delta}_{f,p}(\alpha,\mathbf{M}) \tag{90}$$

□

# References


1. Mages T, Anastasiadi E, Rohner C. Non-Negative Decomposition of Multivariate Information: From Minimum to Blackwell-Specific Information. Entropy. 2024;26(5). doi:10.3390/e26050424.

2. Williams PL, Beer RD. Nonnegative Decomposition of Multivariate Information; 2010. arXiv 1004.2515.

3. Chicharro D, Panzeri S. Synergy and Redundancy in Dual Decompositions of Mutual Information Gain and Information Loss. Entropy. 2017;19(2). doi:10.3390/e19020071.
