# Supplementary material for: Quantifying redundancies and synergies with measures of inequality
Source: PLoS One. 2024 Nov 20;19(11):e0313281. doi: 10.1371/journal.pone.0313281 (PMC11578534; doi:10.1371/journal.pone.0313281)
Supplement: S4 Appendix — Shows the correctness of the suggested implementation for the decomposition in Remark 8 (Section “Decomposing f-inequality”). (PDF) [file pone.0313281.s004.pdf]

S4 Appendix. Implementation suggestion.

This Section demonstrates the correctness of the suggested implementation in Section "Decomposing f-inequality":

1. Assume $\alpha = \top_{\cup}$: The partial contribution of the top element is always zero as shown in Eq (86).

2. Assume $\alpha \neq \top_{\cup}$: Using [1, Lemma 5], the set of immediate successors on the union lattice can be computed as shown in Eq (91) with the function 'dual' of Eq (46).

$$\alpha \neq \top_{\cup}: \qquad \alpha^{+} = \{\alpha \curlyvee \{\mathbf{b}\} \ : \ \mathbf{b} \in \mathrm{dual}(\alpha)\} \tag{91}$$

Using the properties of Eq (85) and Eq (91), we can show the correctness of the suggested implementation by continuing the simplification of Eq (85g):

$$\alpha \neq \top_{\cup}: \qquad I^{\delta}_{f,p}(\alpha,\mathbf{M}) = -I^{\cup}_{f,p}(\alpha,\mathbf{M}) + \sum_{\emptyset \neq \mathbf{B} \subseteq \alpha^{+}} (-1)^{|\mathbf{B}|-1} I^{\cup}_{f,p}(\bigcurlyvee_{\beta \in \mathbf{B}} \beta, \mathbf{M}) \tag{92a}$$

$$\alpha \neq \top_{\cup}: \qquad I^{\delta}_{f,p}(\alpha,\mathbf{M}) = -I^{\cup}_{f,p}(\alpha,\mathbf{M}) + \sum_{\emptyset \neq \mathbf{B} \subseteq \{\alpha \curlyvee \{\mathbf{b}\} \,:\, \mathbf{b} \in \mathrm{dual}(\alpha)\}} (-1)^{|\mathbf{B}|-1} I^{\cup}_{f,p}(\bigcurlyvee_{\beta \in \mathbf{B}} \beta, \mathbf{M}) \tag{92b}$$

$$\alpha \neq \top_{\cup}: \qquad I^{\delta}_{f,p}(\alpha,\mathbf{M}) = -I^{\cup}_{f,p}(\alpha,\mathbf{M}) + \sum_{\emptyset \neq \mathbf{B} \subseteq \{\{\mathbf{b}\} \,:\, \mathbf{b} \in \mathrm{dual}(\alpha)\}} (-1)^{|\mathbf{B}|-1} I^{\cup}_{f,p}(\bigcurlyvee_{\beta \in \mathbf{B}} (\alpha \curlyvee \beta), \mathbf{M}) \tag{92c}$$

$$\alpha \neq \top_{\cup}: \qquad I^{\delta}_{f,p}(\alpha,\mathbf{M}) = -I^{\cup}_{f,p}(\alpha,\mathbf{M}) + \sum_{\emptyset \neq \beta \subseteq \mathrm{dual}(\alpha)} (-1)^{|\beta|-1} I^{\cup}_{f,p}(\alpha \curlyvee \beta, \mathbf{M}) \tag{92d}$$

$$\alpha \neq \top_{\cup}: \qquad I^{\delta}_{f,p}(\alpha,\mathbf{M}) = \sum_{\beta \subseteq \mathrm{dual}(\alpha)} (-1)^{|\beta|-1} I^{\cup}_{f,p}(\mathrm{reduce}(\subset, \alpha \cup \beta), \mathbf{M}) \tag{92e}$$

Therefore, the suggested implementation is correct for all $\alpha \in \mathcal{A}(n)$.

References

1. Mages T, Anastasiadi E, Rohner C. Non-Negative Decomposition of Multivariate Information: From Minimum to Blackwell-Specific Information. Entropy. 2024;26(5). doi:10.3390/e26050424.
